# Assessing the Longitudinal Associations Between Decision-Making Processes and Attention Problems in Early Adolescence

**DOI:** 10.1007/s10802-023-01148-8

**Published:** 2023-12-16

**Authors:** Thea Wiker, Mads L. Pedersen, Lia Ferschmann, Dani Beck, Linn B. Norbom, Andreas Dahl, Tilmann von Soest, Ingrid Agartz, Ole A. Andreassen, Torgeir Moberget, Lars T. Westlye, Rene J. Huster, Christian K. Tamnes

**Affiliations:** 1 NORMENT, Institute of Clinical Medicine, University of Oslo, Oslo, Norway; 2 PROMENTA Research Center, Department of Psychology, University of Oslo, Oslo, Norway; 3 Division of Mental health and Substance Abuse, Diakonhjemmet Hospital, PoBox 23 Vinderen, Oslo, 0319 Norway; 4 K.G. Jebsen Center for Neurodevelopmental Disorders, University of Oslo, Oslo, Norway; 5 Centre for Psychiatry Research, Department of Clinical Neuroscience, Karolinska Institutet & Stockholm Health Care Services, Stockholm Region, Sweden; 6 Division of Mental Health and Addiction, Institute of Clinical Medicine, NORMENT, Oslo University Hospital, University of Oslo, Oslo, Norway; 7 Department of Psychology, University of Oslo, Oslo, Norway; 8 Multimodal Imaging and Cognitive Control Lab, Department of Psychology, University of Oslo, Oslo, Norway; 9 Cognitive and Translational Neuroscience Cluster, Department of Psychology, University of Oslo, Oslo, Norway

**Keywords:** Attention problems, Development, Decision-making, Drift-diffusion modelling, Latent change score modelling, Longitudinal

## Abstract

Cognitive functions and psychopathology develop in parallel in childhood and adolescence, but the temporal dynamics of their associations are poorly understood. The present study sought to elucidate the intertwined development of decision-making processes and attention problems using longitudinal data from late childhood (9–10 years) to mid-adolescence (11–13 years) from the Adolescent Brain Cognitive Development (ABCD) Study (n = 8918). We utilised hierarchical drift-diffusion modelling of behavioural data from the stop-signal task, parent-reported attention problems from the Child Behavior Checklist (CBCL), and multigroup univariate and bivariate latent change score models. The results showed faster drift rate was associated with lower levels of inattention at baseline, as well as a greater reduction of inattention over time. Moreover, baseline drift rate negatively predicted change in attention problems in females, and baseline attention problems negatively predicted change in drift rate. Neither response caution (decision threshold) nor encoding- and responding processes (non-decision time) were significantly associated with attention problems. There were no significant sex differences in the associations between decision-making processes and attention problems. The study supports previous findings of reduced evidence accumulation in attention problems and additionally shows that development of this aspect of decision-making plays a role in developmental changes in attention problems in youth.

**Supplementary Information:**

The online version contains supplementary material available at 10.1007/s10802-023-01148-8.

Accuracy and speed on many experimental tasks of cognition improve over the course of development, but such observable task performance measures are unable to identify the cause of such improvement. That is, slower reaction times (RTs) can reflect both cautious responding or difficulty choosing (Cai et al., 2021). Utilising individual-level parameters from computational models may aid interpretation of individual differences in cognition and cognitive development.

For example, drift-diffusion modelling conceptualises two-alternative, forced choice decision-making as a noisy accumulation-to-bound process (Ratcliff, 1978), and provides four computational parameters based on the observed accuracy and RT: drift rate (v), decision threshold (a), non-decision time (t), and starting point bias. Drift rate reflects the rate of evidence accumulation, in which higher drift rate indicates faster, more accurate decisions. Decision threshold describes response caution or the speed-accuracy trade-off where a wider threshold reflects a more cautious response style. Non-decision time describes perceptual processes like stimulus encoding and motor response, where shorter non-decision time reflects less time spent on encoding and responding. Lastly, the bias parameter indicates whether one choice is preferable of another (Ratcliff & McKoon, 2008). These parameters have been reported to be quite stable across tasks and over shorter time periods (Ratcliff et al., 2010; Schubert et al., 2016).

While improvements in task accuracy and RTs are observed during development, the underlying cognitive decision mechanisms are not well understood. One of the first studies examining age-related differences in DDM parameters in a cross-sectional sample of 135 8-to-20 year-olds reported that children had lower drift rate, wider decision threshold, and longer non-decision time relative to young adults (Ratcliff et al., 2012). This indicates slower information extraction, a more cautious response style, and longer non-decision processes in children. While there is a general lack of studies in children, a large online study of 1.2 million participants aged 10 to 80 replicated these age-related patterns (von Krause et al., 2022). Recent studies have also investigated sex differences and found that females have higher scores on all the DDM parameters (Epstein et al., 2022; von Krause et al., 2022). However, studies are scarce and longitudinal designs examining individual differences in developmental changes are lacking.

Furthermore, drift rate is slower in several mental disorders, including schizophrenia, bipolar disorder, and ADHD (Heathcote et al., 2015; Sripada & Weigard, 2021) and has also been linked to poor self-regulation and impulsivity (Cai et al., 2021; Karalunas & Huang-Pollock, 2013; Smith & Ratcliff, 2009; Sripada & Weigard, 2021; Ziegler et al., 2016). Both poor self-regulation and impulsivity are central in mental disorders, and especially in ADHD (Chamorro et al., 2012; Moeller et al., 2001; Moffitt et al., 2011), which is one of the most studied disorders in relation to drift rate (Cai et al., 2021; Huang-Pollock et al., 2012, 2017, 2020; Karalunas et al., 2012; Karalunas & Huang-Pollock, 2013; Weigard et al., 2016; Weigard & Huang-Pollock, 2014; Ziegler et al., 2016). Additionally, drift rate has shown stronger association with psychopathology symptom scales and task contingencies than conventional metrics like accuracy and RT (Huang-Pollock et al., 2017; Sripada & Weigard, 2021; Ziegler et al., 2016).

Regarding decision threshold in attention problems and ADHD, findings are less consistent than for drift rate (Mowinckel et al., 2015; Ziegler et al., 2016). While some studies report a narrower decision threshold in children with ADHD relative to healthy controls (Weigard & Huang-Pollock, 2014), most studies find no group differences (Cai et al., 2021; Weigard et al., 2016). Similarly, the findings for non-decision time are inconclusive. While some studies report longer non-decision time in ADHD (Cai et al., 2021; Weigard & Huang-Pollock, 2014), possibly reflecting impaired motor speed and coordination (Pitcher et al., 2003; Rommelse et al., 2009), others report no group differences (Weigard et al., 2016). However, relatively few studies report on decision threshold and non-decision time, thereby emphasising the need for more research on these parameters in general and in relation to attention problems, specifically.

While studies report reduced drift rate in ADHD, and that drift rate is a potential trait-like measure, large-scale, longitudinal, population-based studies investigating the development of DDM parameters over time and in relation to attention problems dimensionally are needed. This can provide more insight into the developmental interplay of specific cognitive computational processes and psychopathology (Pedersen et al., 2022). Because attention problems are commonly observed across several mental health disorders, as well as in the general population, it is important to study it dimensionally and transdiagnostically (Lewinsohn et al., 2004; White, 2015; World Health Organization, 1993). Another limitation of previous research is the lack of focus on sex differences. While most studies investigating DDM parameters in individuals with attention problems have controlled for sex (Cai et al., 2021; Karalunas et al., 2012; Karalunas & Huang-Pollock, 2013; Sripada & Weigard, 2021), studies have not explored sex differences in the associations between decision-making processes and attention problems, despite clear indications of such differences in DDM parameters and attention problems separately (Dalsgaard et al., 2020; Lewinsohn et al., 2004; von Krause et al., 2022).

The present study sought to elucidate how DDM parameters develop during early adolescence and how they co-develop with attention problems. To this end, we utilised the Adolescent Brain Cognitive Development (ABCD) Study baseline and two-year follow-up data, consisting of children and adolescents aged 8–14, and latent change score modelling, a powerful and flexible tool within the structural equation modelling (SEM) framework (Kievit et al., 2018; McArdle & Hamagami, 2001). We used multigroup univariate latent change score (ULCS) models to separately estimate average change, variance in change, and if change is dependent on the initial measurement in males and females in the DDM parameters drift rate, decision threshold, and non-decision time estimated from a stop-signal task (SST), and additionally for parent-reported attention problems. Next, we used multigroup bivariate latent change score (BLCS) models to test to which degree the DDM parameters and attention problems correlate at baseline, attention problems at baseline predict change in the DDM parameters, the DDM parameters at baseline predict change in attention problems, and whether changes in the DDM parameters and attention problems co-occur.

For the ULCS models, we hypothesised that drift rate would increase with age, and that decision threshold, non-decision time, and attention problems would decrease with age. Based on the substantial variability shown by von Krause et al. (2022), we also expected individual differences in both change and baseline levels of all DDM measures. However, based on the lack of reporting of sex differences in previous studies, no hypotheses were formulated regarding sex differences in any of the DDM parameters. Conversely, based on Dalsgaard et al. (2020) we anticipated sex differences and individual differences in both baseline level and change in attention problems. Specifically, we expected larger individual differences and a steeper decrease in attention problems for males.

Based on existing literature (Cai et al., 2021; Huang-Pollock et al., 2012, 2017, 2020; Karalunas et al., 2012; Karalunas & Huang-Pollock, 2013; Weigard et al., 2016; Weigard & Huang-Pollock, 2014; Ziegler et al., 2016), for the BLCS models we predicted that drift rate would be negatively associated with attention problems, that drift rate and attention problems at baseline would predict change in attention problems and drift rate, respectively, and finally that the change in both variables would correlate.

## Methods

### Sample

We used the baseline and 2-year follow-up data from the ABCD Study (https://abcdstudy.org/) (Jernigan & Brown, 2018; Volkow et al., 2018). The total sample consists of 11,878 8-11-year-old children at baseline recruited through schools near 21 study sites in the United States (Garavan et al., 2018). Additional information about the sample is provided in the Supplementary materials.

For inclusion in the analyses, all participants were required to have data on both DDM parameters and attention problems on at least one timepoint. Following data cleaning and quality control (see details below), the final sample in our analyses consisted of 8918 participants (4252 females) of which all had baseline measurements and 8005 had follow-up data. At baseline the mean age was 9.9 (SD = 0.6, range = 8.9–11.0) and at follow-up it was 12.0 (SD = 0.7, range = 10.6–14.0).

### Covariates of Interest

To account for the high number of participants being related, we randomly selected one dataset per family resulting in 8699 datasets with trial-level SST data from baseline and 6165 datasets from the 2-year follow-up. Additionally, we wanted to control for population stratification (e.g., ethnicity and socioeconomic status) as it can impact both cognition and psychopathology (Alnæs et al., 2020; Huang et al., 2022) and included genetic ancestry factor (GAF) scores (Raj et al., 2014) and household income collected at baseline as covariates. For details on GAF and household income see Supplementary materials. Lastly, we added age at baseline as a covariate to account for overlapping ages in baseline and follow-up and to get results independent of potential age effects. All covariates were centred prior to analyses. As parental education has been shown to be related to child cognition, we tested the robustness of our analyses to the inclusion of this additional covariate (see Supplementary materials).

### Experimental Task

**Fig. 1:**
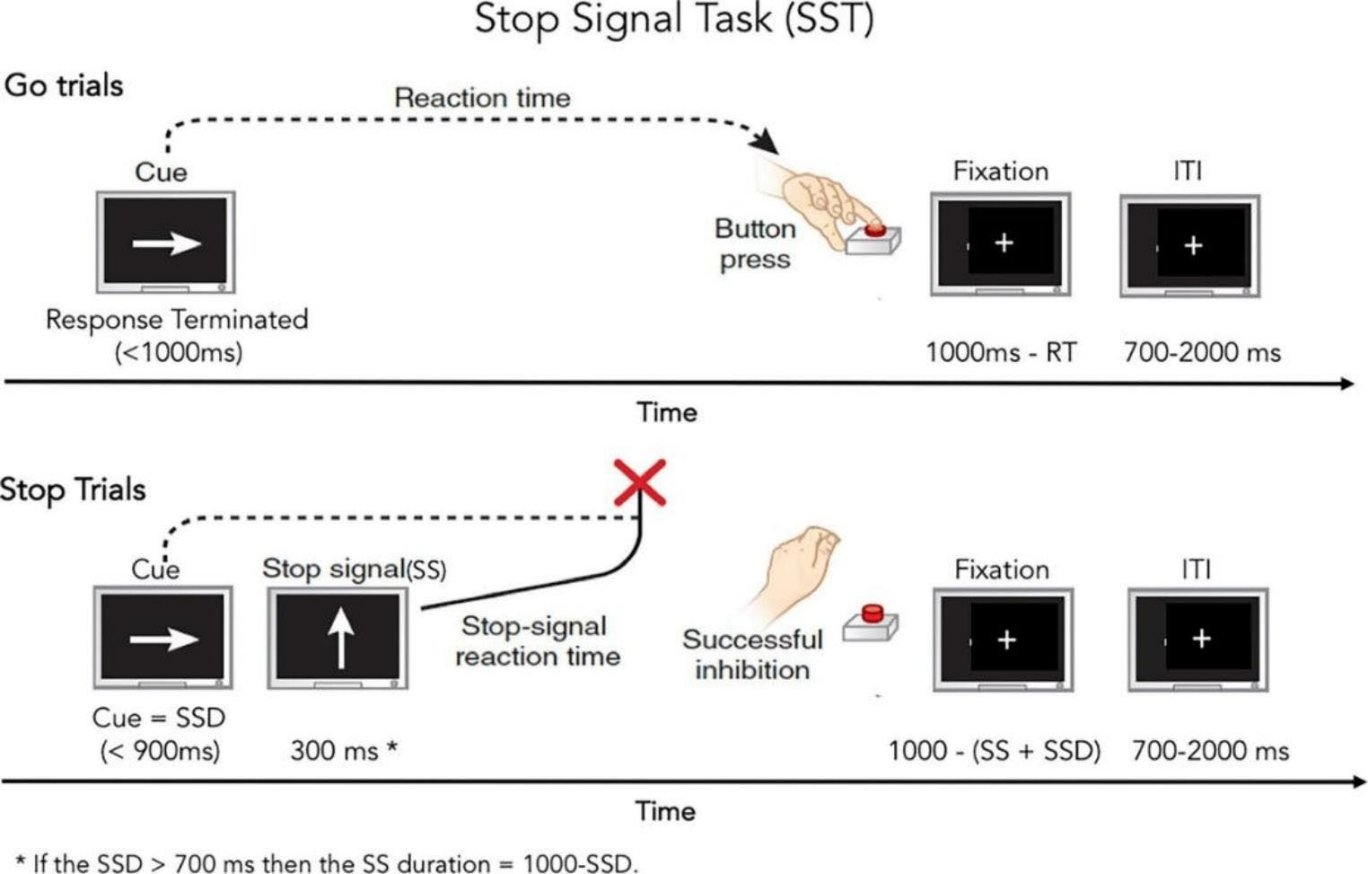
Illustration of the stop-signal task. *Note* ITI = inter-trial interval, RT = reaction time, SSD = stop signal delay, SS = stop-signal, ms = milliseconds. From Casey et al. (2018)

To measure aspects of decision-making, we used trial-level data from a visual SST performed while the participants were in the magnetic resonance imaging (MRI) scanner. In short, the task involves a go stimulus and a stop stimulus. The go stimulus requires a fast response while the occasional stop stimulus on a subset of trials following the go stimulus requires the participant to withhold their response. In the present study, only data from the go trials were included. The task is illustrated in Fig. 1 and described in more detail in the Supplementary materials.

**Fig. 2:**
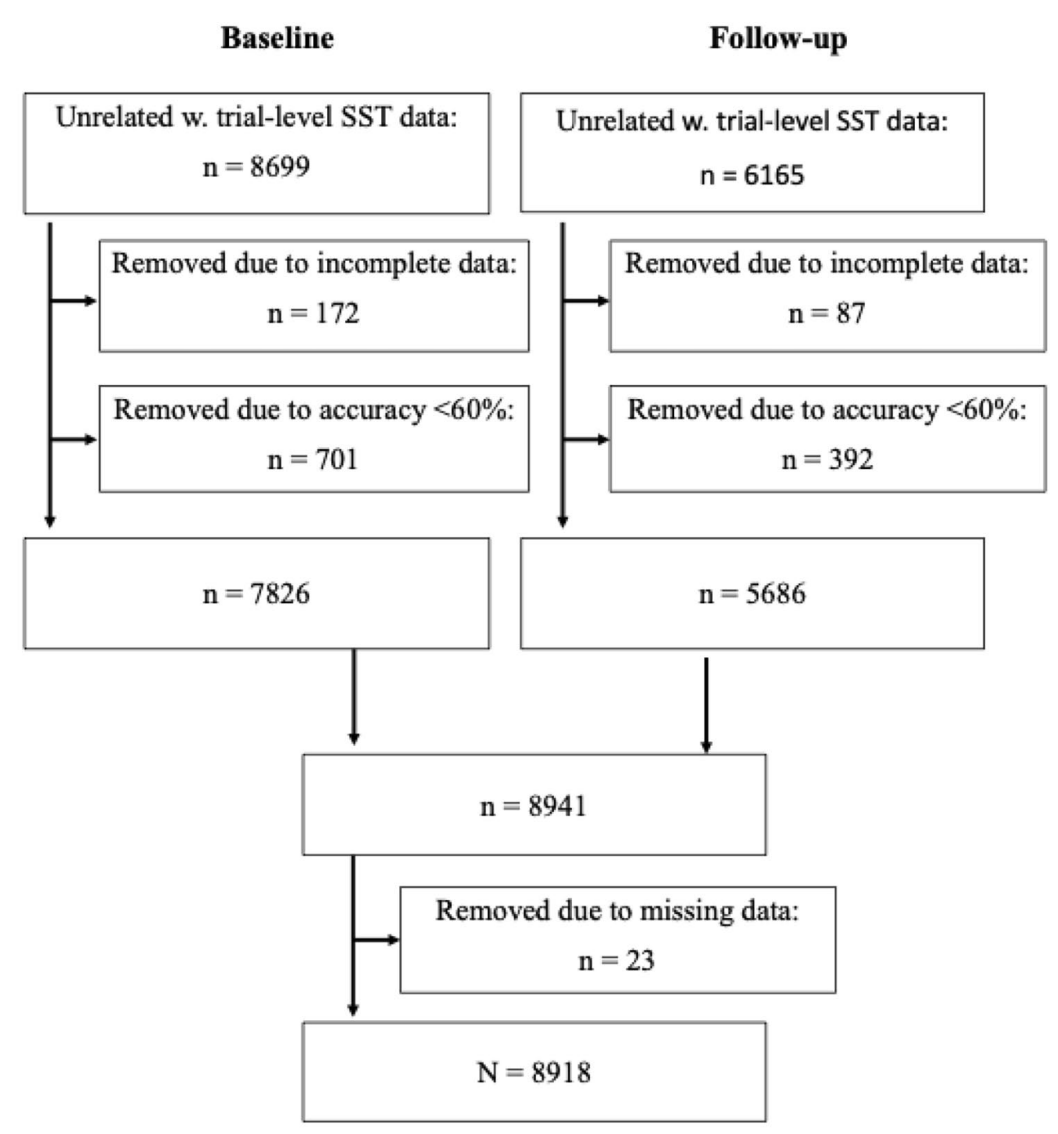
Flow chart of data pre-processing steps

Of the 8699 and 6165 datasets from baseline and follow-up with unrelated subjects and available SST data, 172 and 87 were excluded due to incomplete data, respectively. To ensure task compliance and enough trials, participants with accuracy below 60% on the go trials were excluded (N = 701 for baseline and N = 392 for follow-up). Thus, 7826 datasets from baseline and 5686 datasets from the 2-year follow-up were utilised for estimation of drift-diffusion parameters. Upon merging, the dataset used for DDM parameter estimation consisted of 8941 participants (Fig. 2).

Prior to running the drift-diffusion model, we removed trials with responses faster than 200 ms from each dataset. This resulted in an average of 3.5 go trials removed per participant (SD = 8.1, range = 0-141) and an average of 296.5 go trials retained (SD = 8.1, range = 159–300) for baseline data. For follow-up data, an average of 3.9 trials were removed (SD = 9.0, range = 0-115) and an average of 296.1 trials were retained (SD = 9.0, range = 185–300). For a more detailed overview of number of trials included, see Supplementary Table 1. All data cleaning procedures were performed in R (R Core Team, 2022).

### Drift-Diffusion Modelling

We used the Hierarchical Drift-Diffusion Modelling (HDDM) package in Python (Wiecki et al., 2013) to estimate DDM parameters from choice and RT data from the go trials in the SST (see Supplementary Figure S1). Data was stimulus coded so that rightward responses were coded as 1 and leftward responses as 0. Further, we estimated non-decision time, decision threshold, starting point bias and drift rate for each participant, but allowed the sign of the drift rate to vary depending on condition (left vs. right). To allow the model to account for faster responses for incorrect decisions we also estimated a group parameter for trial-by-trial variation in starting point bias. We considered the direction of the stimulus to not be of interest, and therefore do not report parameter estimates of the starting point bias or its trial-by-trial variability.

As analysing all datasets together in a Bayesian hierarchical model would be too time-consuming, we randomly assigned datasets to one of 80 groups that were estimated separately. For each group, three chains with 3000 samples each were estimated with the first 2000 being discarded as burn-in. This was done to allow the sampling process to identify the region of best-fitting values in the parameter space. To ensure reliable estimates, we used the Gelman-Rubin statistic (Gelman & Rubin, 1992) to test convergence and removed all parameters with rhat values above 1.1 (see Supplementary Table S1 for n removed per DDM parameter). DDM parameters derived from a sufficient amount of trials have been reported with good reliability (r > .7) (Lerche et al., 2017) and the model parameters have been found to be quite stable across different tasks and over a period of eight months (Ratcliff et al., 2010; Schubert et al., 2016). To assess qualitative model fit we ran posterior predictive checks showing that the model could recreate observed choices and RT distributions (see Supplementary Figure S2).

Importantly, there are concerns around entering individual-level parameter estimates from a hierarchical Bayesian model into subsequent null-hypothesis testing analyses as this can potentially bias the test statistics (Boehm et al., 2018; Evans & Wagenmakers, 2019). Separating the sample into 80 groups for the HDDM parameter estimation and using a large sample size may have ameliorated the concerns. However, to make sure our results were unbiased by this issue we performed sensitivity analyses using EZ-diffusion model parameters. The results and interpretations remained largely unchanged (see Supplementary materials).

### Parent-Reported Attention Problems

Attention problems were measured using the attention problems subscale from the Child Behavior Checklist (CBCL, age 6–18 form (Achenbach & Ruffle, 2000). The subscale consists of 10 items describing behaviours that the parents rate on a three-point Likert scale ranging from 0 (not true) to 2 (very true or often true). We used the raw scores from the baseline and 2-year follow-up measurements. The subscale was estimated to have a Cronbach’s alpha of 0.88 for our baseline data and 0.86 for the follow-up data. The subscale correlated r = .93 with the ADHD subscale both at baseline and follow-up in the current sample. However, as the attention problems subscale contains some items that are not specifically related to ADHD (Weigard et al., 2023), we also performed the analyses with the ADHD subscale which resulted in comparable results (see Supplementary materials).

### Statistical Analyses

All data cleaning and statistical analyses were performed in R version 4.2.0 (R Core Team, 2022) and the latent change score models were conducted using the lavaan package version 0.6–11 (Rosseel, 2012) with analysis code available on https://osf.io/tq5ha/. All analyses followed recommendations provided by Kievit et al. (2018). Missing data and non-normality were handled using full information robust maximum likelihood estimation (Enders & Bandalos, 2001). Due to high attrition from baseline to follow-up, we set a requirement that all included participants had data on at least one timepoint for every main variable (i.e., attention problems and the DDM parameters). This resulted in the removal of 23 datasets from the 8941 used for DDM parameter estimation yielding a final sample of 8918 (4252 females, baseline: n = 8918, M_age_=9.9, follow-up: n = 8005, M_age_=12.0). See Fig. 2 for complete overview of exclusions.

**Fig. 3:**
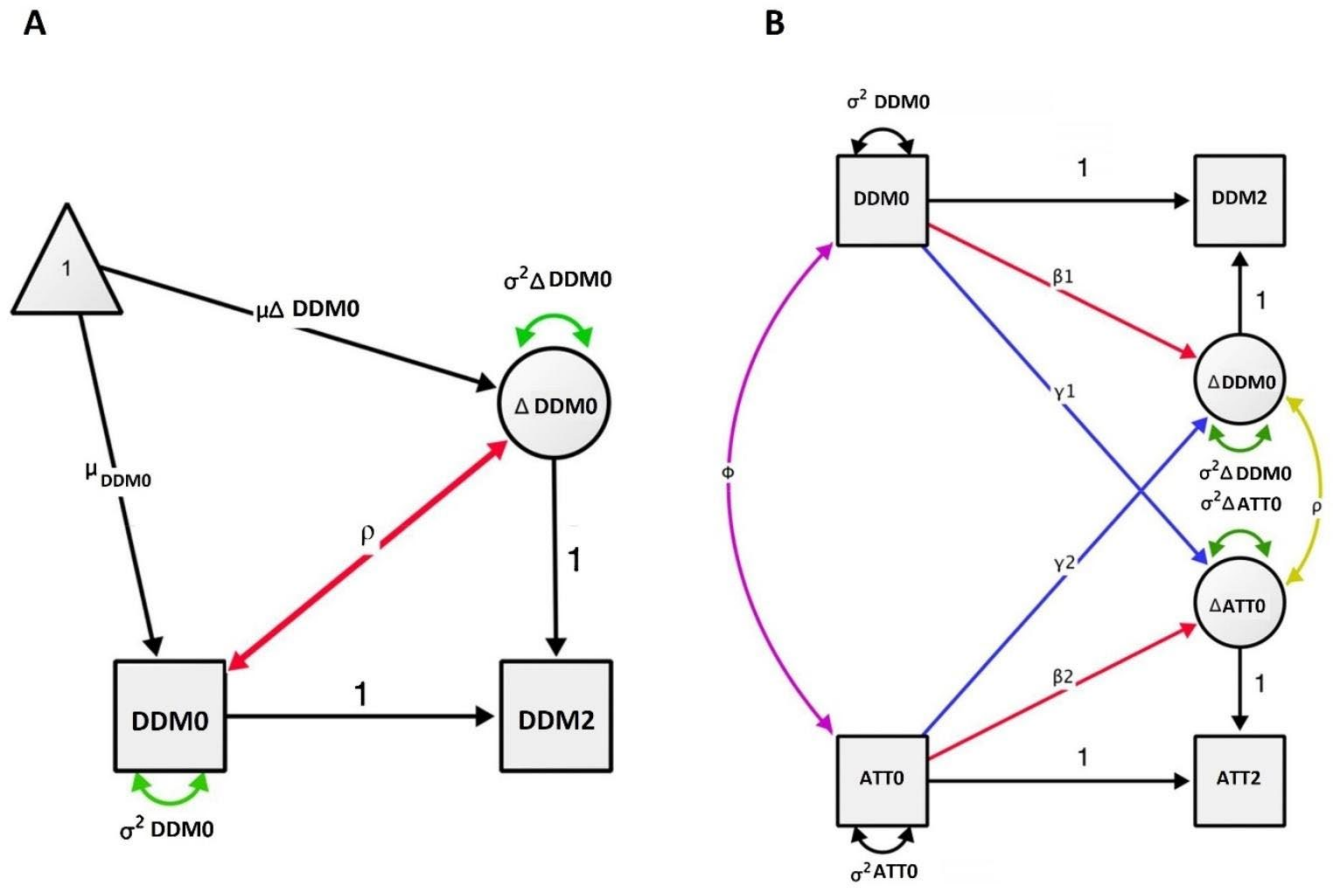
Latent change score models. *Note*: **(A)** Univariate latent change score model illustrating the average change in drift-diffusion parameter (Δ DDM0), variance in change (σ^2^Δ DDM0), and change depending on the initial measurement (red ρ). **(B)** Bivariate latent change score model illustrating correlation between DDM and attention problems (ATT) at baseline (purple φ), regression between DDM and change in ATT (blue γ1), regression between ATT and change in DDM (blue γ2), and co-occurrence of changes in DDM and ATT (yellow ρ). 0 = baseline, 2 = 2-year follow-up. The centred covariates were introduced at the baseline level. Adapted from Kievit et al. (2018)

Four multigroup ULCS models (Kievit et al., 2018; McArdle & Hamigami, 2001) (Fig. 3A) with sex as grouping variable were performed to separately estimate change in the decision-making parameters derived from DDM (i.e., drift rate, decision threshold, and non-decision time), and in attention problems. In each model, a latent change score factor reflecting change from baseline to follow-up was defined. This allowed the estimation of average change, variance in change, and the examination of whether change depends upon the baseline measurement. To estimate sex effects, the models were first run with all parameters unconstrained across sexes. Next, the models were rerun while constraining the model parameters one by one to be equal across sexes. Specifically, we constrained the level of baseline score, variance of the baseline score, level of latent change factor, and variance of the latent change factor. Each model with a constraint was then compared to the initial model without constraints by means of chi-square difference tests. A drop in model fit by constraining provides indication of sex differences on the constrained parameter. All models were controlled for age, GAF, and parental income.

Next, three multigroup BLCS models (Kievit et al., 2018) (Fig. 3B) with sex as grouping variable, one for each of the DDM parameters, were performed to assess the co-development of the DDM parameters and attention problems. The BLCS model builds on the ULCS model and estimates the covariance between baseline measures of e.g., drift rate and attention problems, cross-domain couplings examining whether change in e.g., drift rate is a function of the baseline score of attention problems and vice versa, and correlated change (Kievit et al., 2018). Like in the ULCS models, we constrained the model parameters to test for sex differences by means of chi-square difference tests. Age, GAF, and parental income were included as covariates.

## Results

### Descriptive Statistics

Descriptive statistics for DDM parameters, attention problems, and the covariates age, income and GAF are shown in Supplementary Figures S2-S8. T-tests of sex effects are reported in the Supplementary Table S2. Correlations between all included variables are shown in Supplementary Figures S9-S11 separately for the full sample, females only, and males only.

### Univariate Latent Change Score Models

#### Drift Rate (v)

The multigroup ULCS model for drift rate fit the data well: χ^2^ (12) = 14.495, CFI = 0.999, RMSEA = 0.007, and SRMR = 0.010. The model results are presented in Table 1. The model showed significant positive mean change in drift rate for both females and males, indicating that improvement in evidence accumulation rate occurs from about age 10 to age 12. There were also significant variances in baseline scores and change for both sexes. The significant negative correlation between baseline and change indicated that individuals with high initial levels of drift rate improved less.

When testing for sex differences by means of parameter constraints, the initial level of drift rate (Δχ2 (1) = 34.25, p < .001) and the latent change score (Δχ2 (1) = 7.04, p = .008) differed significantly across sexes, with higher average drift rates and a greater change score for females (estimated mean = 2.74, estimated mean change = 0.434) than males (estimated mean = 2.63, estimated mean change = 0.373). Sex differences were also found for variances of baseline scores (Δχ2 (1) = 5.12, p = .024) with higher variations across participants for males (estimate variance in baseline = 0.674) than females (estimated variance in baseline = 0.625). However, no sex differences were found in the variance of the latent change score (Δχ2 (1) = 1.892, p = .169). The ULCS model on attention problems is visualised in figure S13.

**Table 1:** Results from the multigroup ULCS model on drift rate

Parameter	Females				Males			
	Est.	SE	z	p	Est.	SE	z	p
Baseline mean	2.742	0.014	202.732	**< 0.001**	2.630	0.013	197.057	**< 0.001**
Mean change	0.434	0.017	25.798	**< 0.001**	0.373	0.016	23.017	**< 0.001**
Variance of baseline score	0.625	0.331	27.683	**< 0.001**	0.674	0.016	43.285	**< 0.001**
Variance of change score	0.638	0.234	23.258	**< 0.001**	0.683	0.023	29.408	**< 0.001**
Correlation baseline-change	− 0.414	0.014	-18.221	**< 0.001**	− 0.421	0.015	-19.121	**< 0.001**

*Note.* Est. = unstandardised estimates except for the covariance where standardised estimates are provided, SE = standard error, z = z-score, p = p-value. Significant results are highlighted in bold

#### Decision Threshold (a)

The multigroup ULCS model for decision threshold fit the data well: χ^2^ (12) = 7.21, CFI = 1.00, RMSEA = 0, and SRMR = 0.006. The model results are presented in Table 2. The model demonstrated significant negative mean change in decision threshold for both sexes indicating a decrease in decision threshold from about age 10 to age 12. There were also significant variances in baseline scores and change for both sexes. The significant negative correlations between baseline and change indicate that those with initially high decision threshold decreased more.

When testing for sex differences by means of parameter constraints, the initial level of decision threshold differed significantly across sexes (Δχ2 (1) = 195.41, p < .001), with higher average decision threshold for females (estimated mean = 1.80) than males (estimated mean = 1.62). Sex differences were also found for the variance of the baseline scores (Δχ2 (1) = 8.45, p = .004) with higher variation across female (estimated variance = 0.303) than male participants (estimated variance = 0.252). We further found significant sex differences in mean change (Δχ2 (1) = 3.92, p = .048) with a more negative change for females (estimated change = − 0.12) than males (estimated change = − 0.09). However, no sex differences were found when constraining the variance of the latent change factor (Δχ2 (1) = 0.70, p = .403). The ULCS model on attention problems is visualised in figure S14.

**Table 2:** Results from the multigroup ULCS model on decision threshold

Parameter	Females				Males			
	Est.	SE	z	p	Est.	SE	z	p
Baseline mean	1.799	0.009	189.463	**< 0.001**	1.620	0.008	196.026	**< 0.001**
Mean change	− 0.117	0.012	-9.971	**< 0.001**	− 0.086	0.011	-8.032	**< 0.001**
Variance of baseline score	0.303	0.014	22.274	**< 0.001**	0.252	0.011	22.420	**< 0.001**
Variance of change score	0.332	0.017	19.637	**< 0.001**	0.312	0.016	18.973	**< 0.001**
Correlation baseline-change	− 0.576	0.013	-14.405	**< 0.001**	− 0.530	0.010	-14.393	**< 0.001**

*Note.* Est. = unstandardised estimates except for covariance where standardised estimates are provided, SE = standard error, z = z-score, p = p-value. Significant results are highlighted in bold

#### Non-Decision Time (t)

The multigroup ULCS model for non-decision time fit the data well: χ^2^ (12) = 18.277, CFI = 0.995, RMSEA = 0.012, and SRMR = 0.009. The model results are presented in Table 3. The model demonstrated significant negative mean change in non-decision time for both sexes, indicating a reduction in time spent on non-decision processes from about age 10 to age 12. There were also significant variances in baseline scores and change for both sexes. The negative correlation between baseline and change shows that those with initial high scores decreased more.

When testing for sex differences by means of parameter constraints, the baseline non-decision time differed significantly across sexes (Δχ2 (1) = 83.89, p < .001), with longer non-decision time for females (estimated mean = 0.264) than males (estimated mean = 0.255). Sex differences were also found when constraining the variance of the baseline scores (Δχ2 (1) = 5.60, p = .018) and the variance of the latent change factor (Δχ2 (1) = 6.46, p = .011) with higher variation across participants for males (estimated baseline variance = 0.002 (z-score = 40.91), estimated variance in change = 0.002 (z-score = 29.14)) than females (estimated baseline variance = 0.002 (z-score = 37.57), estimated variance in change = 0.002 (z-score = 27.23). Lastly, constraining the mean level of latent change did not suggest sex differences (Δχ2 (1) = 1.27, p = .261). The ULCS model on attention problems is visualised in figure S15.

**Table 3:** Results from the multigroup ULCS model on non-decision time

Parameter	Females				Males			
	Est.	SE	z	p	Est.	SE	z	p
Baseline mean	0.264	0.001	350.692	**< 0.001**	0.255	0.001	342.234	**< 0.001**
Mean change	.-0.019	0.001	-23.594	**< 0.001**	− 0.021	0.001	-25.917	**< 0.001**
Variance of baseline score	0.002	0.000	37.572	**< 0.001**	0.002	0.000	40.908	**< 0.001**
Variance of change score	0.002	0.000	27.228	**< 0.001**	0.002	0.000	29.142	**< 0.001**
Correlation baseline-change	− 0.592	0.000	-22.755	**< 0.001**	− 0.645	0.000	-26.700	**< 0.001**

*Note.* Est. = unstandardised estimates except for covariance where standardised estimates are provided, SE = standard error, z = z-score, p = p-value. Significant results are highlighted in bold

#### Attention problems

The multigroup ULCS model for attention problems fit the data well: χ^2^ (12) = 18.096, CFI = 0.998, RMSEA = 0.011, and SRMR = 0.006. The model results are presented in Table 4. The model demonstrated significant negative mean change in attention problems for both males and females, indicating a reduction in attention problems from about age 10 to age 12. There were also significant variances in baseline scores and change scores for both sexes. The significant negative correlation between baseline and change indicates that individuals with high initial levels of attention problems tended to decrease more.

When testing for sex differences by means of parameter constraints, the baseline level of attention problems differed significantly across sexes (Δχ2 (1) = 213.77, p < .001), with higher average attention problems for males (estimated mean = 3.50) than females (estimated mean = 2.37). Sex differences were also found for variances of baseline scores (Δχ2 (1) = 77.49, p < .001) and the variance of the latent change factor (Δχ2 (1) = 21.56, p < .001), with higher variations across participants for males (estimated variance in baseline scores = 13.74, estimated variance in change score = 7.06) than females (estimated variance in baseline score = 9.15, estimated variance in change score = 5.44). However, no sex differences in the mean level of latent change were found: Δχ2 (1) = 0.01, p = .907. The ULCS model on attention problems is visualised in figure S16.

**Table 4:** Results from multigroup ULCS model on attention problems

Parameter	Females				Males				
	Est.	SE	z	p	Est.	SE	z	p	
Baseline mean	2.370	0.050	47.683	**< 0.001**	3.501	0.057	61.006	**< 0.001**	
Mean change	− 0.191	0.044	-4.301	**< 0.001**	− 0.243	0.048	-5.118	**< 0.001**	
Variance of baseline score	9.150	0.331	27.683	**< 0.001**	13.743	0.379	36.254	**< 0.001**	
Variance of change score	5.439	0.234	23.258	**< 0.001**	7.059	0.257	27.427	**< 0.001**	
Correlation baseline-change	− 0.444	0.214	-14.617	**< 0.001**	− 0.418	0.245	-16.819	**< 0.001**	

*Note.* Est. = unstandardised estimates except for covariance where standardised estimates are provided, SE = standard error, z = z-score, p = p-value. Significant results are highlighted in bold

## Bivariate Latent Change Score

**Table 5:** Results from BLCS models for each DDM parameter where numbers 1–4 represent the effects of interest: (1) correlation at baseline, (2) regression (attention problems at baseline related to change in DDM parameter), (3) regression (DDM parameter at baseline related to change in attention problems), (4) correlation of change score

	Parameter	Females				Males			
		Est.	SE	z	p	Est.	SE	z	p
Drift rate (v)									
1	att0↔v0 (ϕ)	− 0.244	0.042	-5.769	**< 0.001**	− 0.350	0.052	-6.687	**< 0.001**
2	att0→Δv (γ_1_)	− 0.062	0.005	-2.982	**0.003**	− 0.052	0.004	-2.756	**0.006**
3	v0→Δatt (γ_2_)	− 0.084	0.055	-4.447	**< 0.001**	− 0.033	0.057	-1.858	0.063
4	Δatt↔Δv (ρ)	− 0.127	0.035	-3.671	**< 0.001**	− 0.108	0.038	-2.829	**0.005**
Threshold (a)									
1	att0↔a0 (ϕ)	− 0.016	0.029	− 0.948	0.343	− 0.003	0.032	− 0.162	0.871
2	att0→Δa (γ_1_)	0.003	0.003	0.197	0.843	− 0.012	0.003	− 0.717	0.474
3	a0→Δatt (γ_2_)	− 0.020	0.079	-1.051	0.293	− 0.010	0.100	− 0.532	0.594
4	Δatt↔Δa (ρ)	− 0.038	0.020	-1.895	0.058	− 0.004	0.027	− 0.189	0.850
Non-decision time (t)									
1	att0↔t0 (ϕ)	− 0.015	0.002	− 0.850	0.395	− 0.014	0.003	− 0.812	0.417
2	att0→Δt (γ_1_)	0.015	0.000	0.791	0.429	− 0.008	0.000	− 0.518	0.604
3	t0→Δatt (γ_2_)	− 0.044	1.007	-2.303	**0.021**	− 0.021	1.064	-1.147	0.251
4	Δatt↔Δt (ρ)	− 0.034	0.002	-1.443	0.149	− 0.027	0.002	-1.314	0.189

**Note.** 0 = baseline measure, est. = estimates, SE = standard error, z = z-score, p = p-value, Δ = change. All reported effects are standardised. Statistically significant findings are highlighted in bold

### Drift Rate(v)

**Fig. 4:**
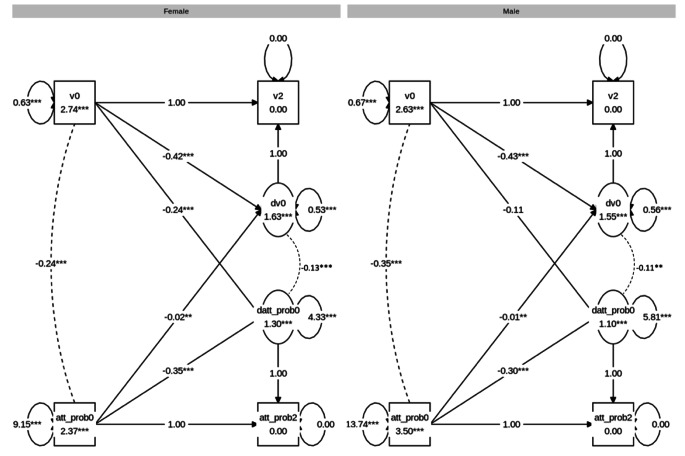
Graph showing the multigroup BLCS models for drift rate (v) and attention problems (att_prob). *Note*. The estimates are unstandardised

First, a multigroup BLCS model for attention problems and drift rate was modelled and the fit was good: χ^2^ (24) = 32.319, CFI = 0.999, RMSEA = 0.009, SRMR = 0.009. Inspection of the four parameters of interest, reflecting the four possible attention problems-drift rate relationships (see Table 5), showed a statistically significant negative correlation between attention problems and drift rate at baseline for both males (*r* = − .12, *p* < .001) and females (*r* = − .24, *p* < .001). The results also showed statistically significant associations of correlated change for both males (*r* = − .11, *p* = .005) and females (*r* = − .13, *p* < .001). The negative correlations indicate that those with greater increase in drift rate over time showed a greater decrease in attention problems. There were additional coupling effects in which attention problems at baseline predicted change in drift rate for males (β=-0.05, p = .006) and females (β = − 0.06, p = .003), and drift rate at baseline predicted change in attention problems for females (β=-0.08, p < .001) but not males (β=-0.03, p = .063). Next, χ^2^-difference testing for sex differences in the coefficients were carried out. Here, no significant difference between males and females were found for any of the four attention problems-drift rate relationships. The results are visualised in Fig. 4.

### Decision Threshold (a)

**Fig. 5:**
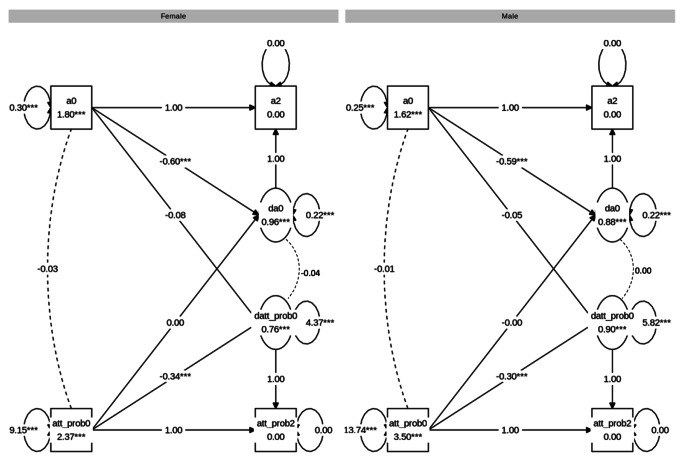
Graph showing the multigroup BLCS models for decision threshold **(a)** and attention problems (att_prob). *Note*. The estimates are unstandardised

Second, a BLCS model for attention problems and decision threshold was modelled and the fit was good: χ^2^ (23) = 25.572, CFI = 0.999, RMSEA = 0.005, SRMR = 0.007. Inspection of the four parameters of interest, reflecting the four possible attention problems-decision threshold relationships (see Table 5), showed no statistically significant correlation between attention problems and decision threshold at baseline for males or females. The results also showed no statistically significant associations of correlated change. Lastly, there were no significant coupling effects. Moreover, χ^2^-difference tests indicated no significant differences between males and females for any of the four attention problems-decision threshold relationships. The results are visualised in Fig. 5.

### Non-Decision Time (t)

**Fig. 6:**
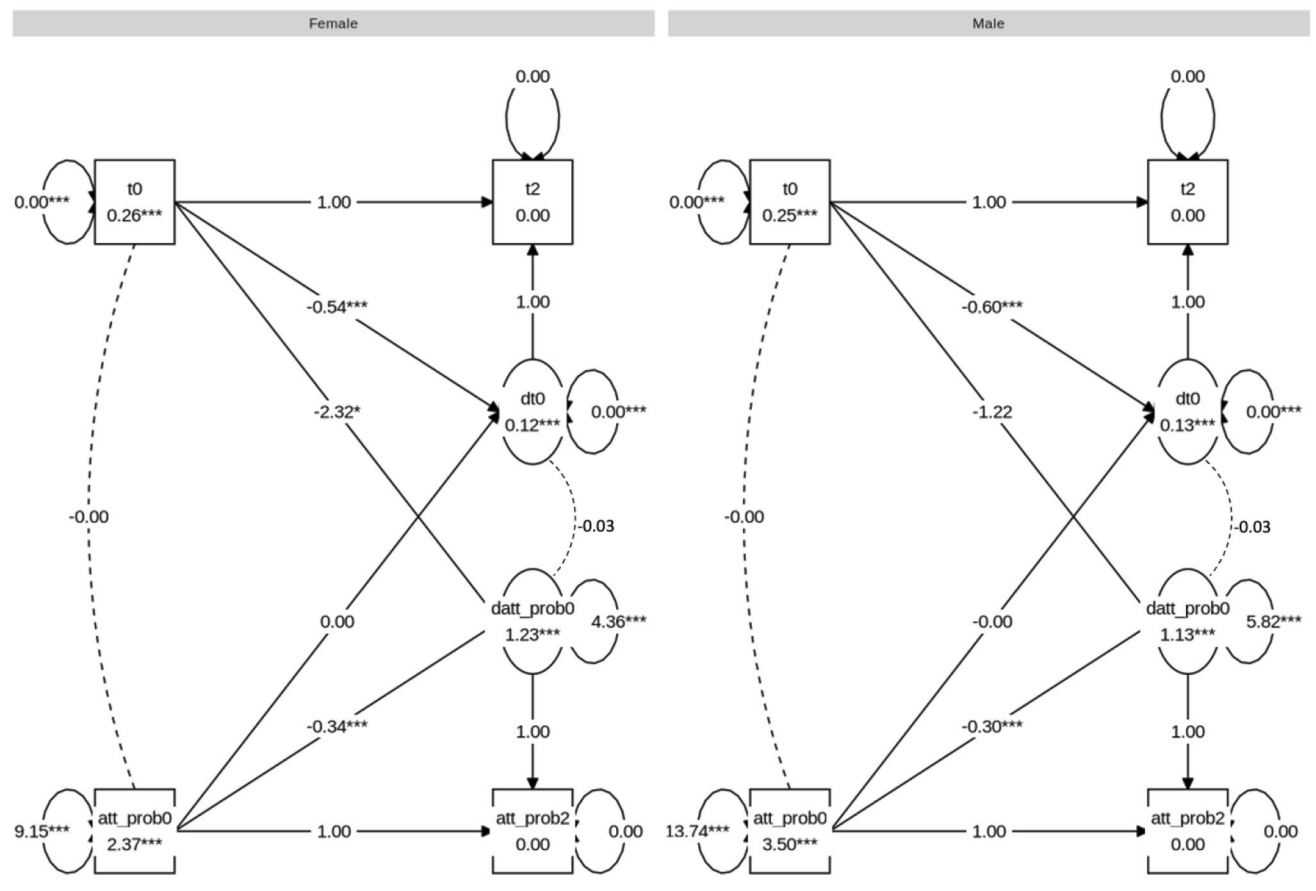
Graph showing the multigroup BLCS models for non-decision time (t) and attention problems (att_prob). *Note*. The estimates are unstandardised

Third, a BLCS model for attention problems and non-decision time was modelled and the fit was good: χ^2^ (24) = 38.285, CFI = 0.997, RMSEA = 0.012, SRMR = 0.009. Inspection of the four parameters of interest, reflecting the four possible attention problems-non-decision time relationships (see Table 5), showed no statistically significant correlation between attention problems and non-decision time at baseline for males or females. The results further showed no statistically significant associations of correlated change. Lastly, there were no significant coupling effects except for non-decision time at baseline predicting change in attention problems for females (β=-0.04, p = .021). Next, χ^2^-difference testing for sex differences were carried out. Here, no significant differences between males and females were found for any of the four attention problems-non-decision time relationships. The results are visualised in Fig. 6.

## Discussion

In this study, we investigated the co-development of decision-making processes and attention problems using multigroup latent change score modelling in a large population-based longitudinal sample spanning the age range 8–14. Our main findings add to the existing literature by showing reduced evidence accumulation (drift rate; i.e., slower and less accurate responses) in attention problems. We additionally provide novel indications of temporal couplings in the development of drift rate and attention problems across both sexes, and show that evidence accumulation, and not response caution (decision threshold) or encoding- and responding processes (non-decision time), is associated with attention problems.

As hypothesised for the ULCS models, we found that drift rate increased with age, while decision threshold, non-decision time, and attention problems decreased. This indicates more efficient evidence accumulation, less cautious response style, less time spent on non-decision processes like encoding and responding, and a reduction in attention problems from about age 10 to age 12. As expected, we also found variance in both baseline scores and change scores for all variables. Lastly, our models show that change depended on the initial level of the measure with a negative correlation between baseline and change in all models. This negative correlation is commonly observed in developmental studies and may be explained by the notion that individuals with low initial scores have a greater potential to obtain higher scores at subsequent assessments (Rogosa et al., 1982; Von Soest & Hagtvet, 2011).

Although no hypotheses were formulated with regards to sex differences in the DDM parameters, we found that females had higher baseline scores on all variables (Epstein et al., 2022; von Krause et al., 2022). Females also showed greater increase in drift rate, higher variance in the decision threshold baseline score and a larger reduction in decision threshold. Males, on the other hand, showed greater variance in baseline score of drift rate and higher variance in both baseline and change for non-decision time. No sex differences were observed for the variance in the change score of drift rate or decision threshold, or in the change score for non-decision time. Overall, this indicates that females have a more efficient evidence accumulation, a more cautious response style, and spend more time on non-decision processes like encoding and responding. While the analyses on EZ-diffusion parameters were overall comparable to those with HDDM parameters, it is worth noting the significant sex differences. While higher drift rate, threshold and non-decision time is observed for females in both models, the differences in significant sex effects may indicate uncertainty of these results and further analyses are needed.

Finally, for attention problems, we anticipated larger individual differences and a steeper decrease in attention problems for males relative to females. However, we only found support for higher variance, as there were no sex differences in the change score. Yet, we did find higher baseline scores for males. Overall, this indicates that males and females in this sample do not differ in the degree of reduction of attention problems, even though they differ in the baseline scores. As we used a dimensional approach to attention problems rather than a categorical, diagnostic approach, our results apply to the general population including subclinical symptomatology and attention deficits across categorical mental disorders (American Psychiatric Association, 2022; Racer & Dishion, 2012).

As hypothesised, the multigroup BLCS model on drift rate and attention problems revealed a significant negative association at baseline in line with previous studies indicating less efficient evidence accumulation in youth with attention problems (Heathcote et al., 2015; Sripada & Weigard, 2021; White et al., 2010, 2016). Further, the model also showed that the change scores for drift rate and attention problems were negatively correlated, i.e., as drift rate increases, attention problems decrease, albeit with a small effect size. In terms of coupling effects, we as expected found that attention problems at baseline negatively predicted the level of change in drift rate, also with small effect size. This indicates that high levels of attention problems at baseline predict less improvement in the efficiency of evidence accumulation. However, while we hypothesised that drift rate would predict the change in attention problems, this was only true for females. Yet, none of the associations revealed significant sex differences.

For the multigroup BLCS models on decision threshold and non-decision time, no hypotheses were formulated due to lack of robust previous findings (Cai et al., 2021; Mowinckel et al., 2015; Weigard et al., 2016; Ziegler et al., 2016). It was therefore unsurprising that the current model on decision threshold and attention problems revealed no significant relationships and no significant sex differences. Similarly, the model on non-decision time and attention problems showed no significant sex differences, however non-decision time at baseline significantly negatively predicted change in attention problems in females but not males. The null findings might be attributable to the age of the current sample as von Krause et al. (2022) found that at around age 18, the decision threshold parameter begins increasing rather than slightly decreasing. Similarly, non-decision times are fastest at around the age of the current sample. It would therefore be interesting to see how these parameters develop later in adolescence and potentially interact with attention problems.

Overall, the results suggests that evidence accumulation, and not response caution or non-decision processes like encoding and responding, is associated with attention problems in both sexes, and this relationship is also evident longitudinally, though with small effect sizes. Considering the higher prevalence of attention problems in males and the higher scores of females on the DDM parameters as shown in previous research (Dalsgaard et al., 2020; Epstein et al., 2022) and in the present study, the specific associations between drift rate and attention problems in both sexes shown in the multigroup BLCS models are interesting.

It is also interesting to compare the results from the analysis with HDDM-derived parameters and with EZ-derived parameters. The main difference between the results was the somewhat differing significance of sex differences. This may be attributable to small effects and males and females displaying effects in the same direction, only differing slightly in magnitude. This elucidates the importance of considering effect sizes in addition to significance.

The findings presented in the current paper are not without limitations. First, there are design issues with the SST used in the ABCD study (Bissett et al., 2021). Most of the issues are related to stop trials and incorrect calculation of accuracies. As the current study only used the go trials from the task and re-calculated the accuracies based on trial-level data, the impact of the issues should be minimal. However, studies show that when stop-signals are possible, participants have slower RTs (Vink et al., 2015; Zandbelt & Vink, 2010). Considering that the stop-signal probability here is lower than generally recommended (Verbruggen et al., 2019), this should interfere less with the RTs. Second, the consistency and validity of the DDM parameters have been debated (Bompas et al., 2023; Schubert et al., 2016). We therefore recommended that future studies use more than one task to better capture the variance in the estimates (Schubert et al., 2016). Third, and as indicated above, the age range is relatively narrow in the present study, with literature indicating that many developmental processes manifest a more pronounced acceleration a few years later (Ratcliff et al., 2012; von Krause et al., 2022).

Although it was beyond the scope of the present study, in future studies, it would be interesting to investigate to what degree drift rate and the other DDM parameters are associated with other dimensions of psychopathology (e.g., p-factor, internalising, externalising). Relatedly, it would be interesting to see if the present results can be replicated when accounting for potential medications and when including multiple raters of psychopathology, for instance by combining parent- and teacher reports (Cordova et al., 2022). Additionally, the neural mechanisms underlying the DDM parameters in youth are mainly unknown. While some research has been carried out (e.g., (Manning et al., 2021), more specificity in terms of brain regions and mechanisms are needed. Additionally, robustness through validation and replication utilising different cognitive tasks and samples are necessary, especially in light of the small effect sizes in the current study. This will allow further understanding of the contribution of evidence accumulation in the development of attention problems and other behavioural and mental disorders in youth. Lastly, the current study only had two timepoints. Future studies should therefore utilise more timepoints to cover a wider age range and capture the changes in DDM parameters as described in von Krause et al. (2022) and how these changes relate to the development of psychopathology. The addition of more timepoints also allow the estimation of potential non-linear developmental patterns.

In conclusion, the present study investigated the longitudinal coupling of decision-making processes and attention problems in youth. We elucidate the utility of computational modelling to decompose task behaviour and build on the existing literature showing that commonly observed prolonged RTs can be attributed to inefficient evidence accumulation (drift rate), and not response caution (decision threshold) or encoding- and responding mechanisms (non-decision time), in parent-reported attention problems. Furthermore, using longitudinal data and analysis, the study showed that change in drift rate and attention problems are predicted by the baseline measure of the other. Importantly, these findings were found across females and males, indicating comparable developmental patterns and similar decision-making impairments in attention problems regardless of sex. While computational psychiatry still has limited direct clinical implications (Hauser et al., 2019; Karvelis et al., 2023), it is a promising avenue for improving our understanding of cognition in psychopathology.

### Electronic Supplementary Material

Below is the link to the electronic supplementary material.

Supplementary Material 1
