# Supplementary material for: Assessing the Longitudinal Associations Between Decision-Making Processes and Attention Problems in Early Adolescence
Source: Res Child Adolesc Psychopathol. 2023 Dec 16;52(5):803–17. doi: 10.1007/s10802-023-01148-8 (PMC11063004; doi:10.1007/s10802-023-01148-8)
Supplement: Supplementary file 1 — Supplementary Material 1 [file 10802_2023_1148_MOESM1_ESM.docx]

**Supplementary materials**

**Assessing the longitudinal associations between decision-making processes and parent-reported attention problems in early adolescence**

Thea Wiker, Mads L. Pedersen, Lia Ferschmann, Dani Beck, Linn B. Norbom, Andreas Dahl, Tilmann von Soest, Ingrid Agartz, Ole A. Andreassen, Torgeir Moberget, Lars T. Westlye, Rene J. Huster, Christian K. Tamnes

**Contents**

Supplementary methods p. 4

Sample p. 4

Covariates of interest p. 4

Experimental task p. 4

Table S1: Descriptive statistics of number of trials included in the
DDM estimation p. 5

Drift-diffusion modelling p. 5

Figure S1: Illustration of the drift-diffusion model p. 5

Table S2. Number of datasets converted to NA due to poor
convergence of Gelman-Rubin statistic >1.1 p. 6

Figure S2. Response time distribution model fit p. 6

Supplementary results p. 7

Descriptive statistics p. 7

Figure S3. Distribution of the drift-diffusion model parameter
 drift rate p. 7

Figure S4. Distribution of the drift-diffusion model parameter
 decision threshold p. 8

Figure S5. Distribution of the drift-diffusion model parameter
 non-decision time p. 8

Figure S6. Distribution of attention problems p. 9

Figure S7. Distribution of age at baseline p. 10

Figure S8. Distribution of parental income at baseline p. 10

Figure S9. Distribution of genetic ancestry at baseline p. 11

Table S3. T-tests on sex differences in variables of interest p. 11

Figure S10. Correlations between behavioural variables for
 the full sample p. 12

Figure S11. Correlations between behavioural variables for
 the females p. 13

Figure S12. Correlations between behavioural variables for
the males p. 14

ULCS p. 15

Figure S13. Graph showing results from the ULCS model on
 drift rate (v) p. 15

Figure S14. Graph showing results from the ULCS model on
 decision threshold (a) p. 15

Figure S15. Graph showing results from the ULCS model on
 non-decision time (t) p. 16

Figure S16. Graph showing results from the ULCS model on
 attention problems (att_prob) p. 16

Results from latent change score models with parental education as covariate p. 16

ULCS p. 17

Table S4. Fit statistics for the multigroup ULCS models p. 17

Drift rate (v) p. 17

Table S5. Results from the multigroup ULCS model on drift rate p. 17

Decision threshold (a) p. 18

Table S6. Results from the multigroup ULCS model on decision
threshold p. 18

Non-decision time (t) p. 18

Table S7. Results from the multigroup ULCS model on non-
decision time p. 19

Attention problems p. 19

Table S8. Results from the multigroup ULCS model on attention
problems p. 19

BLCS p. 20

Table S9. Fit statistics for the multigroup BLCS models p. 20

Table S10. Results from BLCS models for each DDM parameter p. 20

Drift rate (v) p. 20

Decision threshold (a) p. 21

Non-decision time (t) p. 21

Results from latent change score models with EZ-diffusion model parameters p. 22

ULCS p. 22

Table S11. Fit statistics for the multigroup ULCS models p. 22

Drift rate (v) p. 22

Table S12. Results from the multigroup ULCS model on drift rate p. 23

Decision threshold (a) p. 23

Table S13. Results from the multigroup ULCS model on decision
threshold p. 23

Non-decision time (t) p. 23

Table S14. Results from the multigroup ULCS model on non-
decision time p. 24

BLCS p. 24

Table S15. Fit statistics for the multigroup BLCS models p. 24

Table S16. Results from BLCS models for each DDM parameter p. 24

Drift rate (v) p. 25

Decision threshold (a) p. 25

Non-decision time (t) p. 26

Results from latent change score models with the ADHD subscale p. 26

ULCS p. 27

Table S17. Results from the multigroup ULCS model on attention
problems p. 27

BLCS p. 27

Table S18. Fit statistics for the multigroup BLCS models p. 27

Table S19. Results from BLCS models for each DDM parameter p. 27

Drift rate (v) p. 28

Decision threshold (a) p. 28

Non-decision time (t) p. 28

**Supplementary methods**

**Sample**

All procedures were approved by a central Institutional Review Board (IRB) at the University of California, San Diego, and in some cases by individual site IRBs. Informed consent was obtained from a parent or legal guardian, and children provided assent. The current study has been approved by the Regional Committees for Medical and Health Research Ethics (REK 2019/943).

We utilized the annual curated data release 4.0 for all data. All ABCD Study data is stored in the NIMH Data Archive Collection #2573, which is available for registered and authorized users (Request #7474, PI: Westlye). The data will be permanently available as a persistent dataset defined in the NDA Study 1299 (release 4.0). NDA Study 1299 has been assigned the DOI 10.15154/1523041.

**Covariates of interest**

The GAF scores are four continuous variables labelled African, American, European, and East Asian describing proportion of genetic ancestry. The scores are part of the tabulated data from the ABCD and are calculated based on the Bayesian clustering results with 1000 genome reference panel. Household income was computed by taking the average of parental income, partner income, and combined income. The income variable consisted of values 1-10 representing different income intervals: 1 = Less than $5,000; 2 = $5,000 through $11,999; 3 = $12,000 through $15,999; 4 = $16,000 through $24,999; 5 = $25,000 through $34,999; 6 = $35,000 through $49,999; 7 = $50,000 through $74,999; 8 = $75,000 through $99,999; 9 = $100,000 through $199,999; 10 = $200,000 and greater.

**Experimental task**

In the stop-signal task, the participants were presented with a white arrow pointing left or right on a black background (the go stimulus) and asked to respond with their dominant hand on a two-button response pad based on the direction of the arrow (Casey et al., 2018). On a subset of trials, here 1/6, the go stimulus was followed by a stop stimulus: an arrow pointing upwards. On these trials, the participants were instructed to withhold their response. The go arrow was presented on the screen for 1000 milliseconds (ms) or until a response was made. Total trial duration was set to 1000 ms, and a fixation cross was presented on the screen following the go arrow when a response was made. On stop trials, the go arrow was presented for the duration of the stop-signal delay (SSD; the interval between the go stimulus and the stop stimulus) or until a response was made. The stop arrow was then presented for 300 ms. However, if the SSD was more than 700 ms, the stop stimulus was presented for 1000 ms minus the SSD. As the maximum SSD was 900 ms, the minimum stop signal duration was 100 ms. The inter-trial interval varied between 700 ms and 2000 ms and the stimulus onset asynchrony thus ranged between 1700 ms and 3000 ms. To ensure approximately 50% accuracy rate on stop trials, the SSD was staircased such that a successful inhibition trial increased the SSD by 50 ms and a failed inhibition trial reduced it by 50 ms. The starting SSD was 50 ms. The task consisted of two runs of 180 trials each, for a total of 360 trials (300 go trials, 60 stop trials), and each run lasted 349 seconds (≈6 minutes) each.

Following quality control as described in main paper, drift-diffusion modelling was performed. For an overview of N trials submitted to DDM, see Table S1.

**Table S1.**Descriptive statistics of number of trials included in the DDM estimation.

| Measure (trials) | Mean | SD | Range |
| --- | --- | --- | --- |
| NgoAcc0 | 258.59 | 29.09 | 103-300 |
| NgoErr0 | 22.59 | 15.97 | 0-114 |
| NgoOm0 | 15.33 | 17.40 | 0-113 |
| NgoAcc2 | 266.11 | 27.96 | 127-300 |
| NgoErr2 | 19.76 | 15.44 | 0-103 |
| NgoOm2 | 10.23 | 14.69 | 0-111 |
| %goAcc0 | 87.10 | 8.67 | 59.73-100 |
| %goErr0 | 7.70 | 5.60 | 0-38 |
| %goOm0 | 5.20 | 5.93 | 0-37.67 |
| %goAcc2 | 89.75 | 8.0 | 59.78-100 |
| %goErr2 | 6.77 | 5.5 | 0-38.19 |
| %goOm2 | 3.48 | 5.01 | 0-37 |

**Drift-diffusion modelling**

**
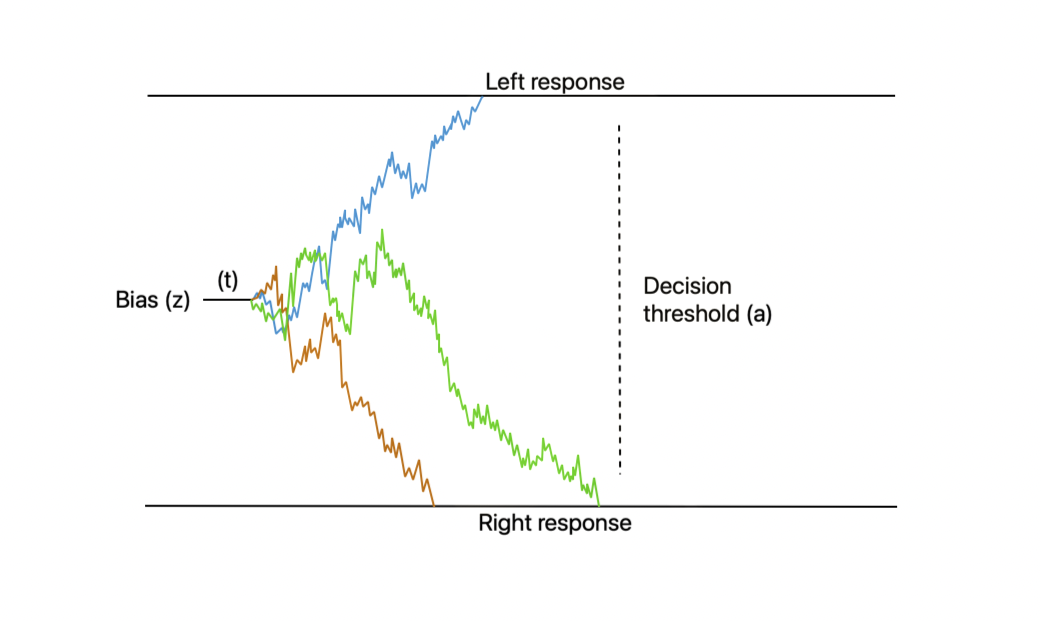
**

**Figure S1.** Illustration of the drift-diffusion model.

**Table S2.**Number of datasets converted to NA due to poor convergence of Gelman-Rubin statistic > 1.1.

| Parameter | N removed |
| --- | --- |
| a0 | 13 |
| v0 | 6 |
| t0 | 26 |
| a2 | 16 |
| v2 | 6 |
| t2 | 15 |

Note. 0 = baseline, 2 = follow-up, a = threshold, v = drift rate, t = non-decision time

**
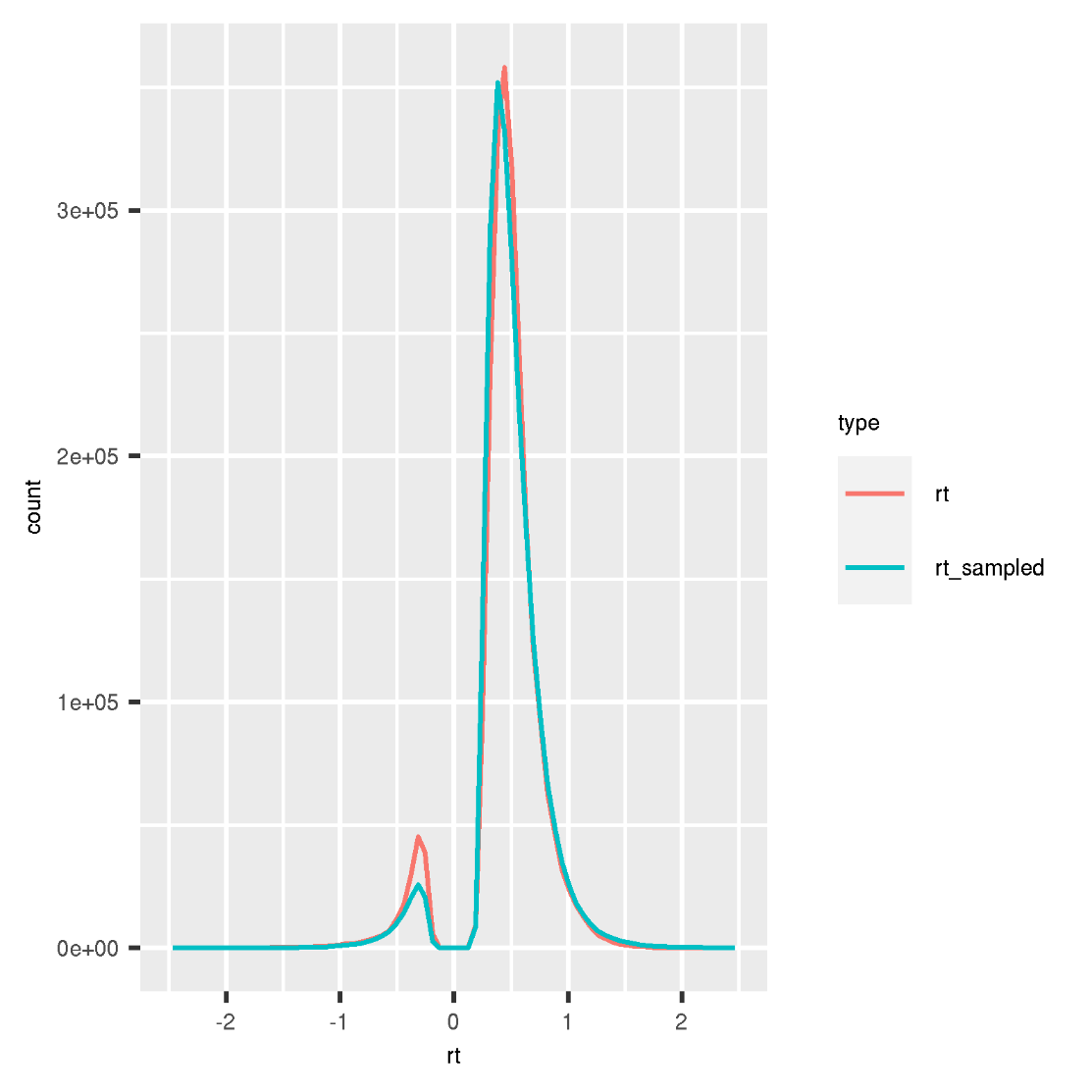
**

**Figure S2.** Response time distribution model fit. Observed (pink) and predicted (blue) response time distributions for go trials in the stop-signal task. Response times on incorrect trials are coded as negative to separate responses for correct and incorrect trials.

**Supplementary results**

**Descriptive statistics**

Descriptive statistics for DDM the parameters drift rate, decision threshold, and non-decision time, attention problems, and the covariates age, income, and GAF are shown separately for males and females in Supplementary Figures S3-S9, respectively. T-tests of sex effects are reported in the Supplementary Table S2. Correlations between all included variables (drift rate, decision threshold, non-decision time, attention problems, age, income, and the four genetic ancestry factor (GAF) scores) are shown in Supplementary Figure S10-S12 separately for the full sample, females only, and males only.


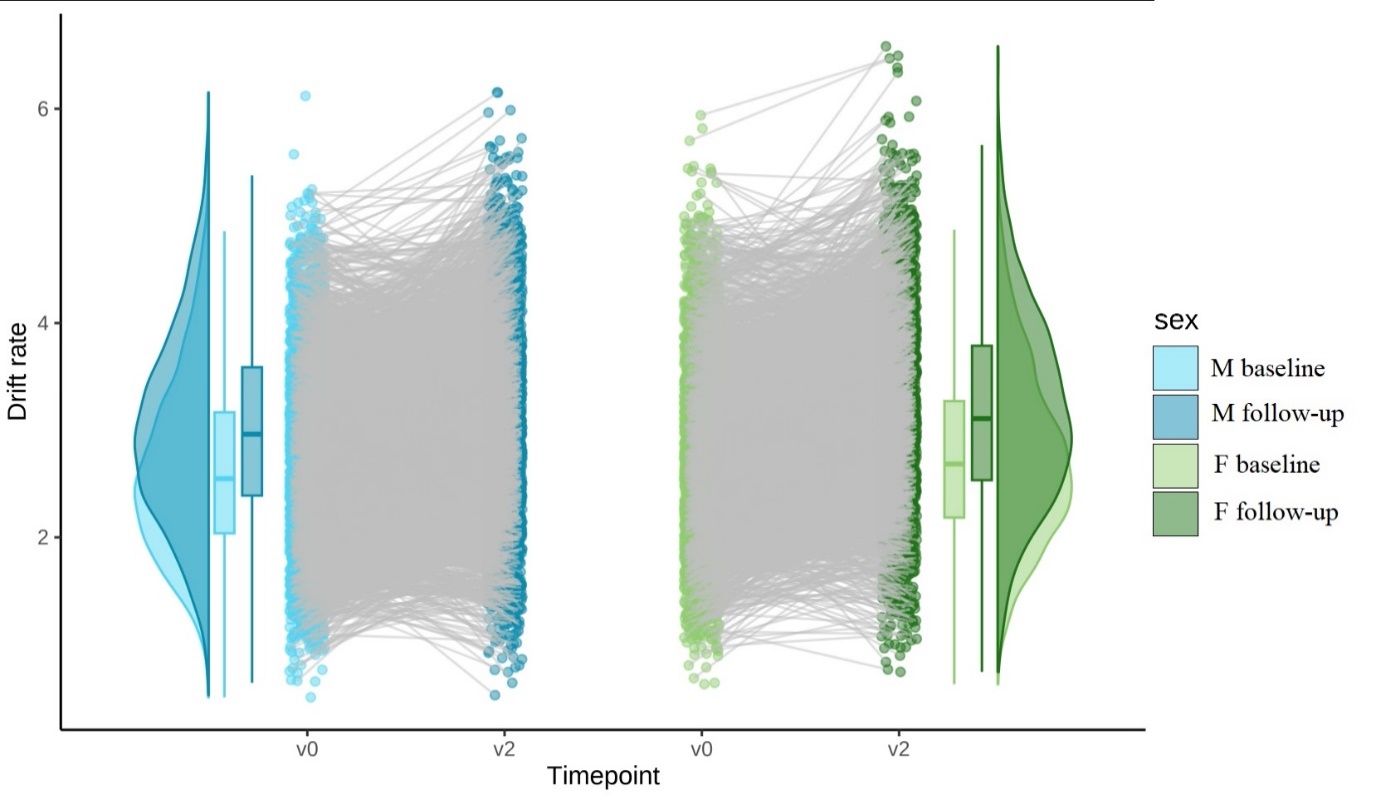


**Figure S3.** Distribution of the drift-diffusion modelling parameter drift rate. Raincloud plot of drift rate grouped by sex (males = blue, females = green) showing change in drift rate from baseline (v0) to follow-up (v2). M = male, F = female.


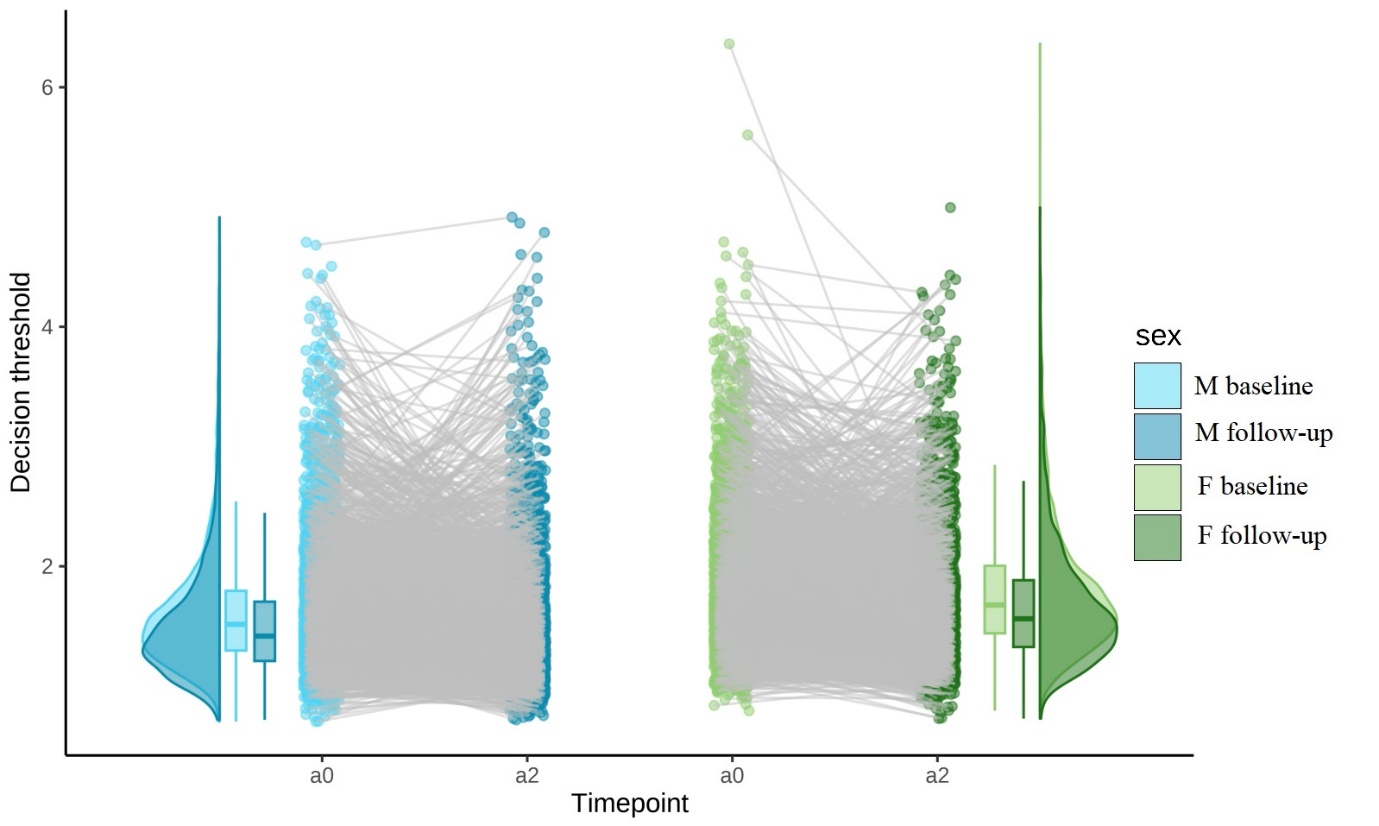


**Figure S4.** Distribution of the drift-diffusion modelling parameter decision threshold. Raincloud plot of decision threshold grouped by sex (males = blue, females = green) showing change in decision threshold from baseline (a0) to follow-up (a2). M = male, F = female.


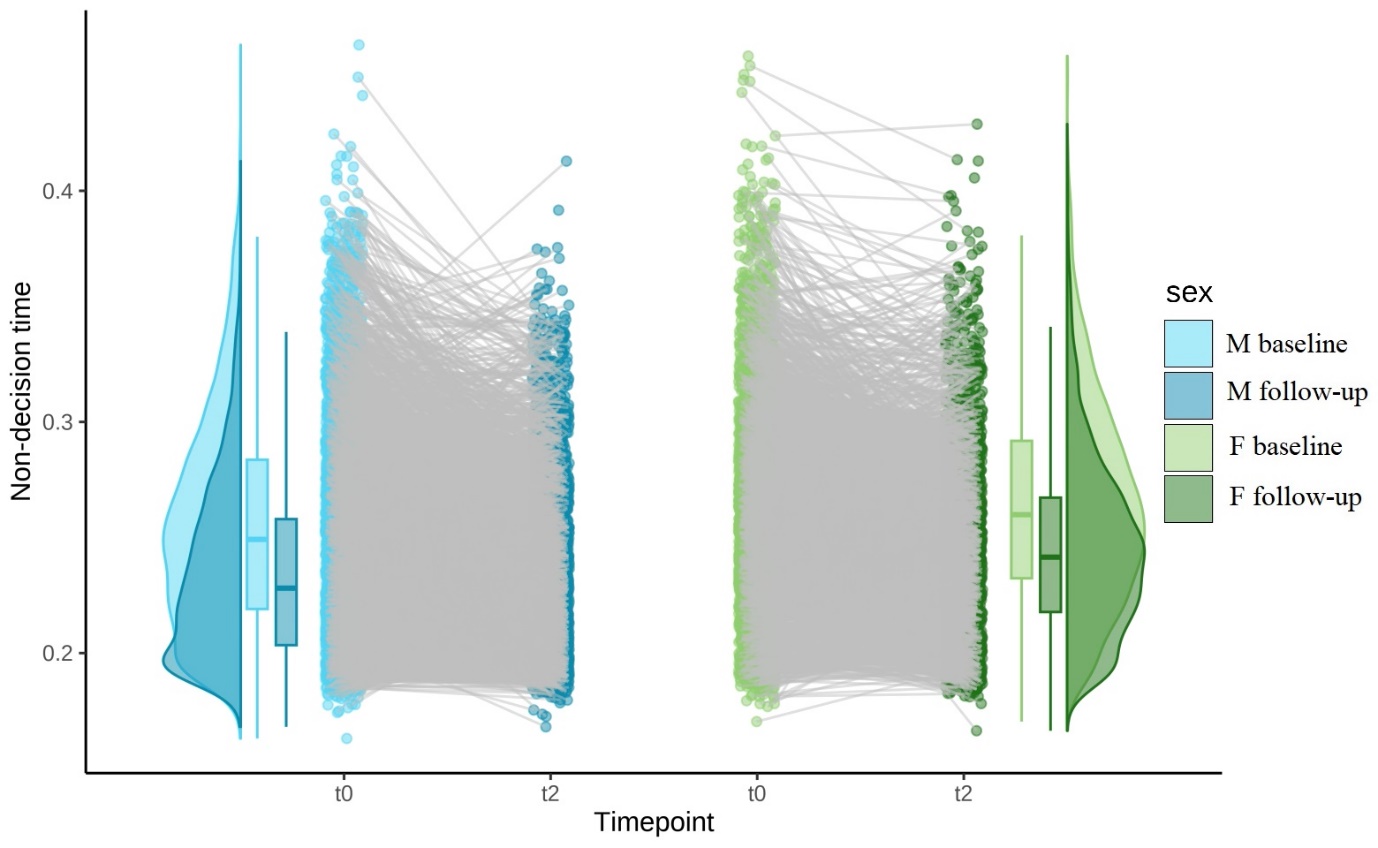


**Figure S5.** Distribution of the drift-diffusion modelling parameter non-decision time. Raincloud plot of non-decision time grouped by sex (males = blue, females = green) showing change in non-decision time from baseline (t0) to follow-up (t2). M = male, F = female.


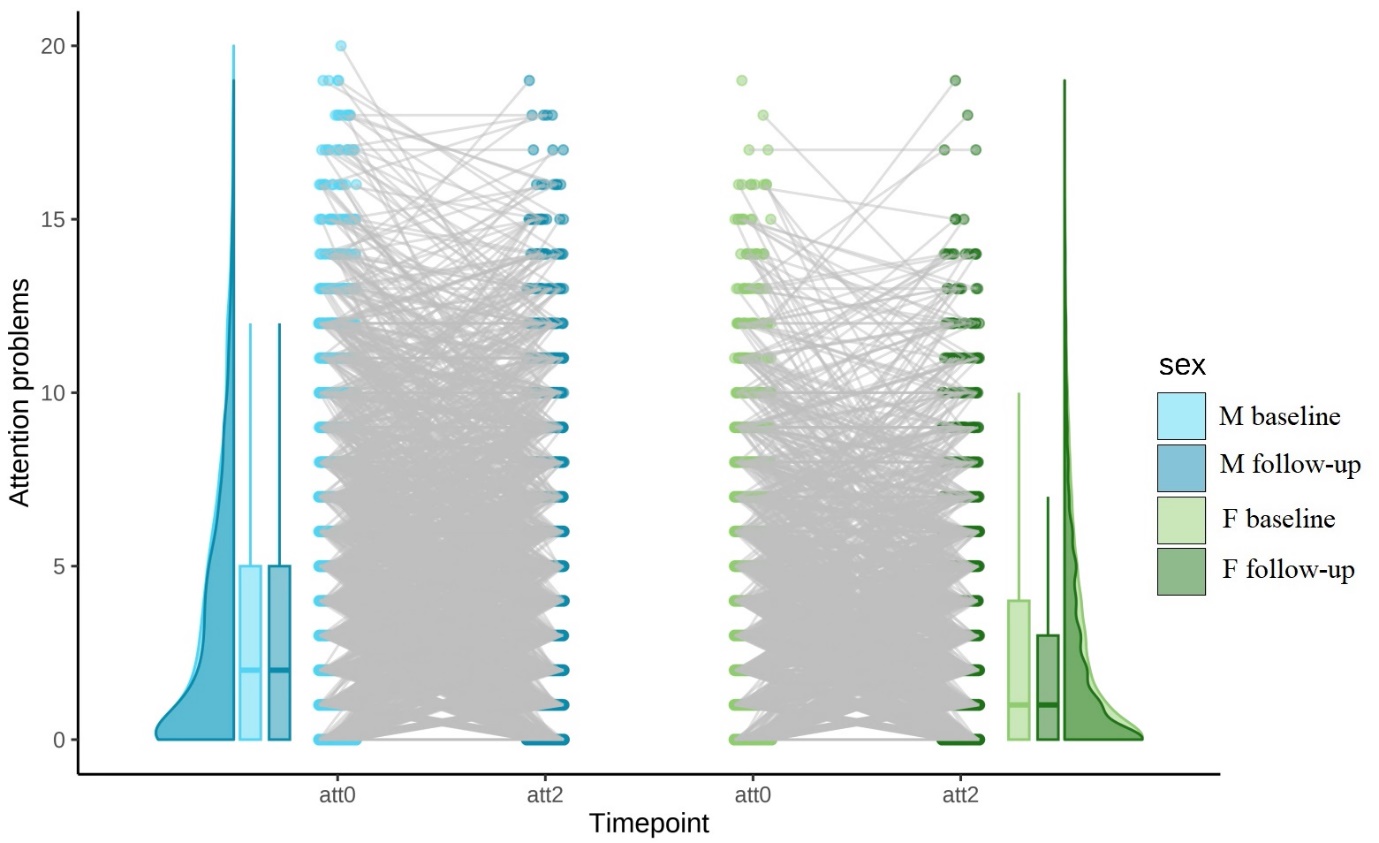


**Figure S6.** Distribution of attention problems. Raincloud plot of attention problems grouped by sex (males = blue, females = green) showing change in attention problems from baseline (v0) to follow-up (v2). M = male, F = female.

**
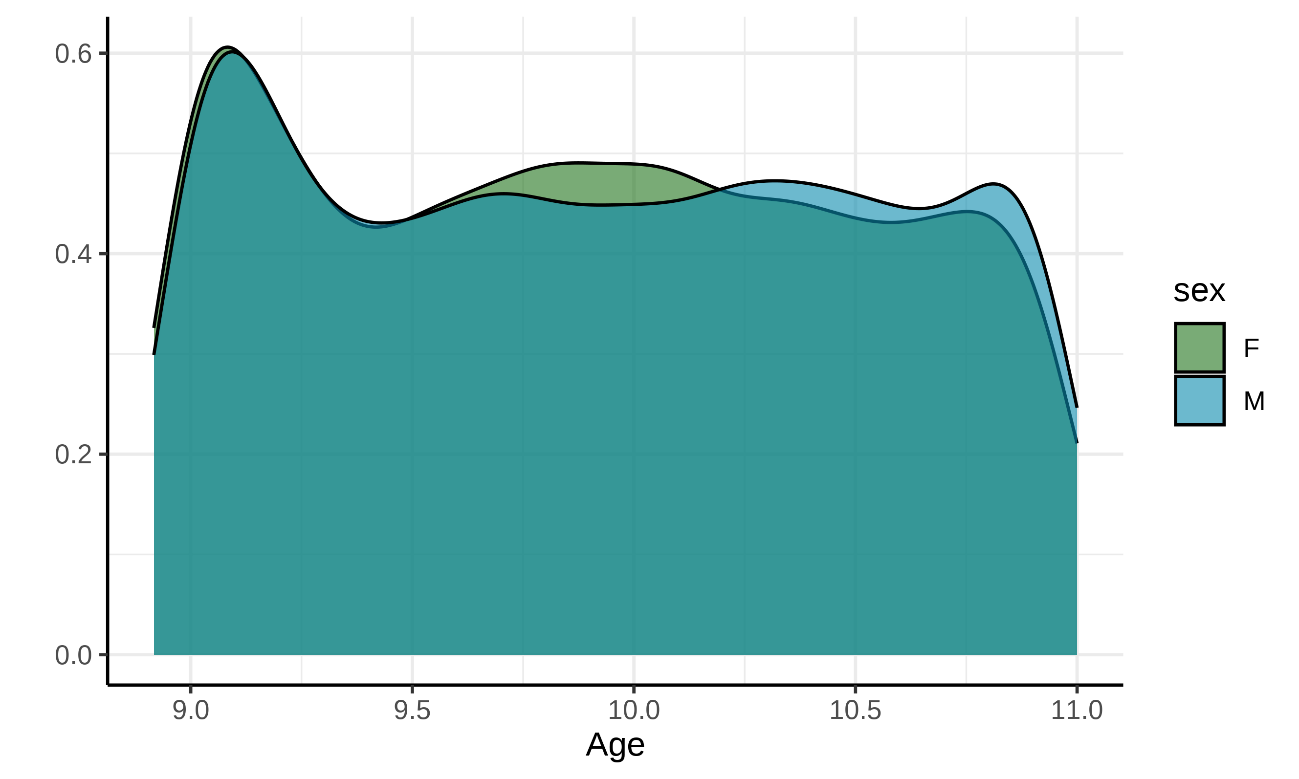
**

**Figure S7.** Distribution of age at baseline. Density plot of age at baseline grouped by sex (males = blue, females = green). M = male, F = female.


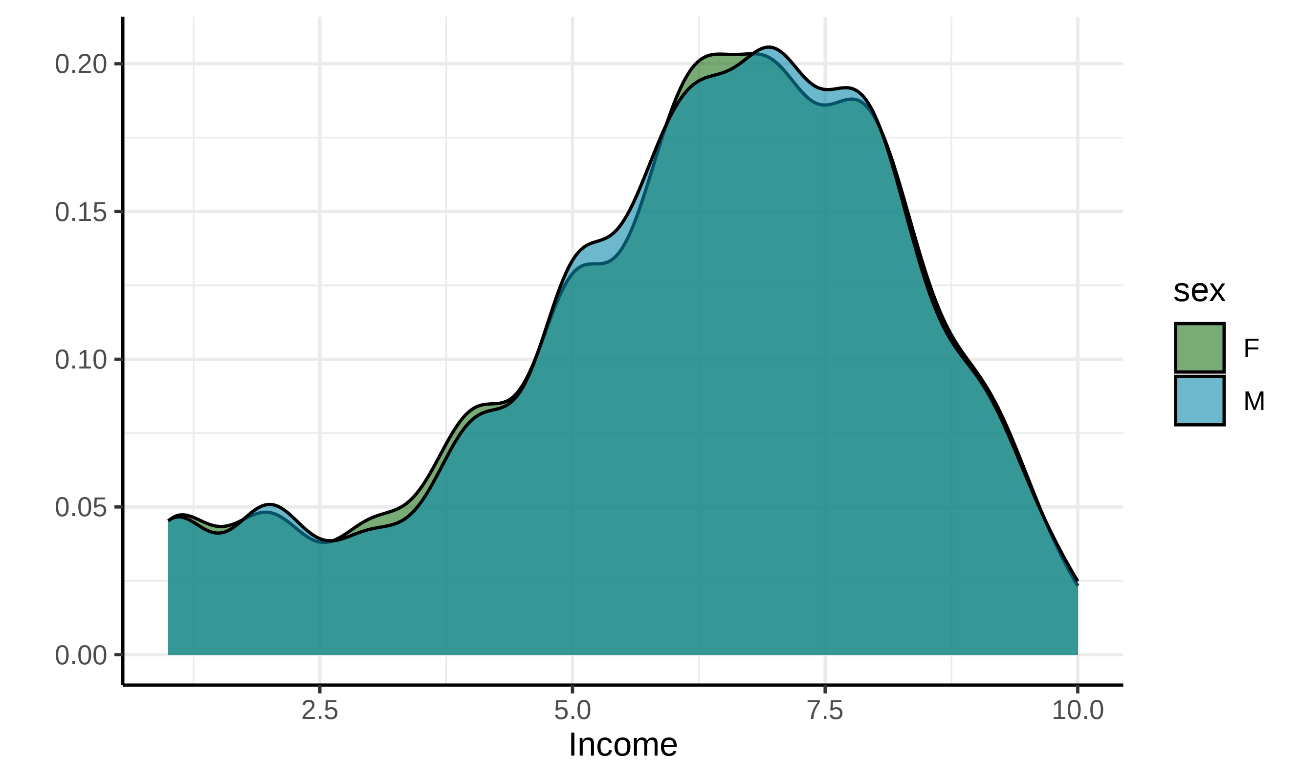


**Figure S8.** Distribution of parental income at baseline. Density plot of parental income at baseline grouped by sex (males = blue, females = green). M = male, F = female.


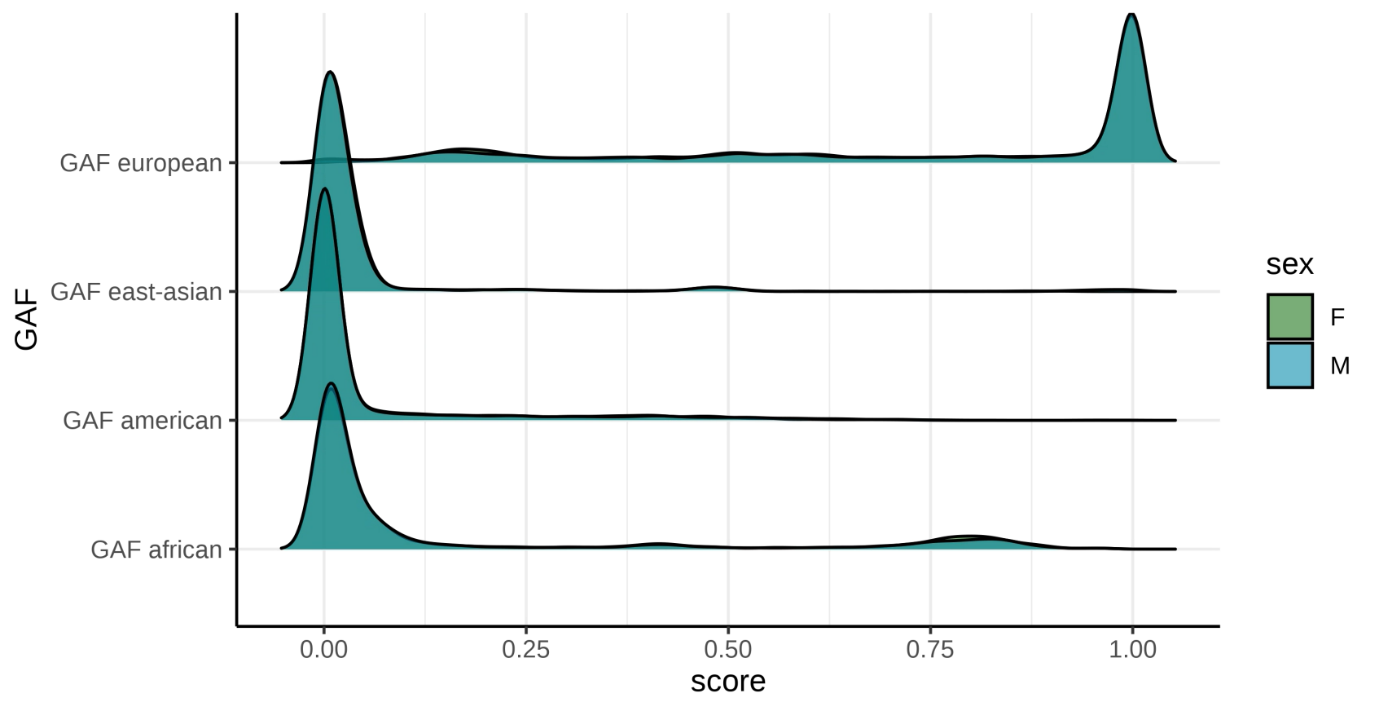


**Figure S9.** Distribution of genetic ancestry at baseline. Density plot of the genetic ancestry factor (GAF) scores at baseline grouped by sex (males = blue, females = green). M = male, F = female.

**Table S3.**T-tests on sex differences in variables of interest

|  | t | p |
| --- | --- | --- |
| Sex-v0 | 6.21 | <.001 |
| Sex-v2 | 6.64 | <.001 |
| Sex-a0 | 14.98 | <.001 |
| Sex-a2 | 10.66 | <.001 |
| Sex-t0 | 9.58 | <.001 |
| Sex-t2 | 10.29 | <.001 |
| Sex-att0 | -14.94 | <.001 |
| Sex-att2 | -12.96 | <.001 |

*Notes. The sex variable was coded as Females = 0, Males = 1, so positive values indicate higher scores for females and negative scores indicates higher scores for males. 0 = baseline measure, 2 = 2-year follow-up measure, p = p-value, v = drift rate, a = threshold, t = non-decision time, att = attention problems.*

*
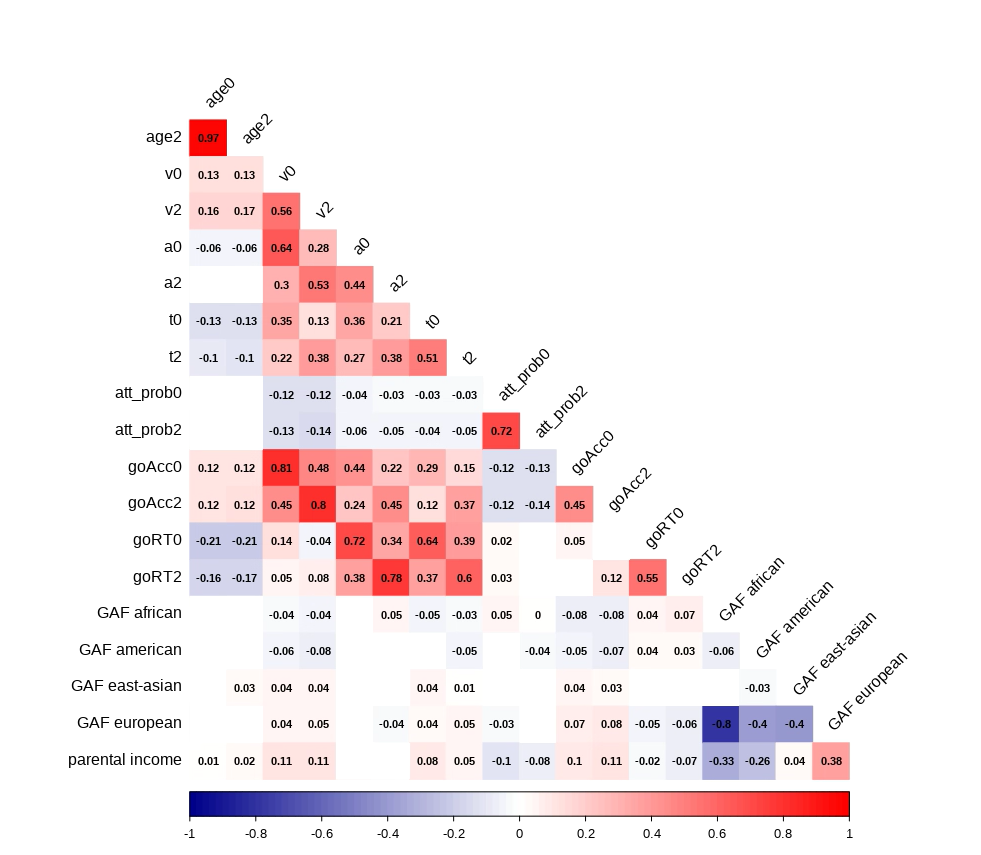
*

**Figure S10.** Correlations between behavioural variables for the full sample. Correlation plot showing the significant correlations between the variables included in our analyses. 0 = baseline measure, 2 = 2-year follow-up measure, r = Pearson’s r, p = p-value, v = drift rate, a = threshold, t = non-decision time, RT = reaction time, acc = accuracy, GAF = genetic ancestry factor.

*
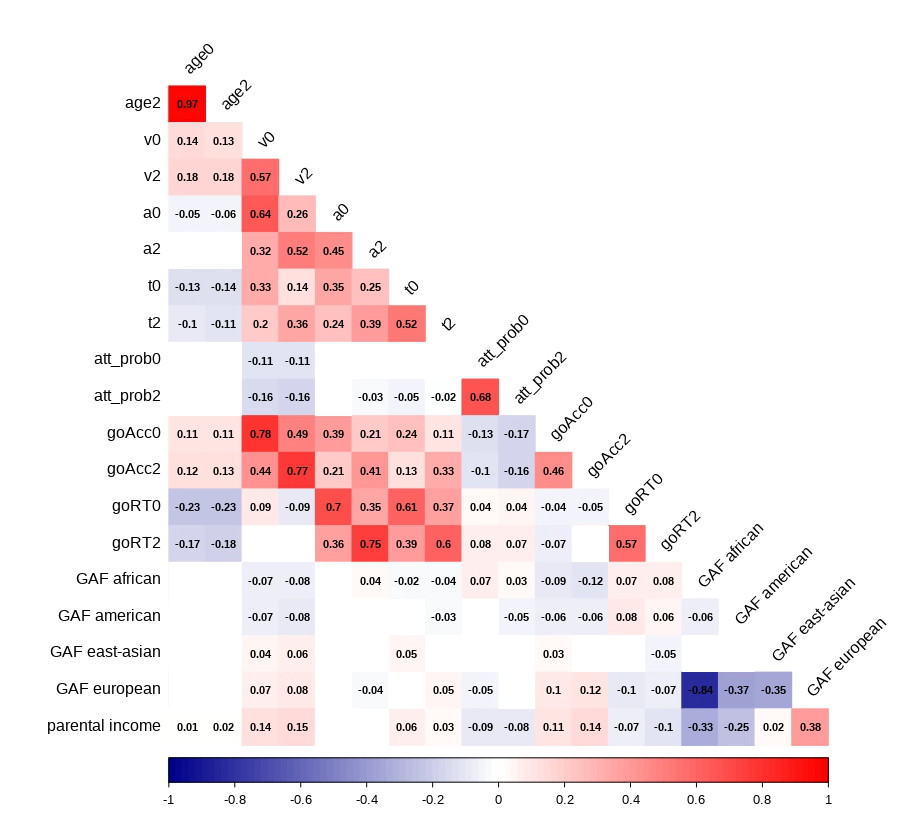
*

**Figure S11.** Correlations between behavioural variables for the females. Correlation plot showing the significant correlations between the variables included in our analyses. 0 = baseline measure, 2 = 2-year follow-up measure, r = Pearson’s r, p = p-value, v = drift rate, a = threshold, t = non-decision time, RT = reaction time, acc = accuracy, GAF = genetic ancestry factor.

*
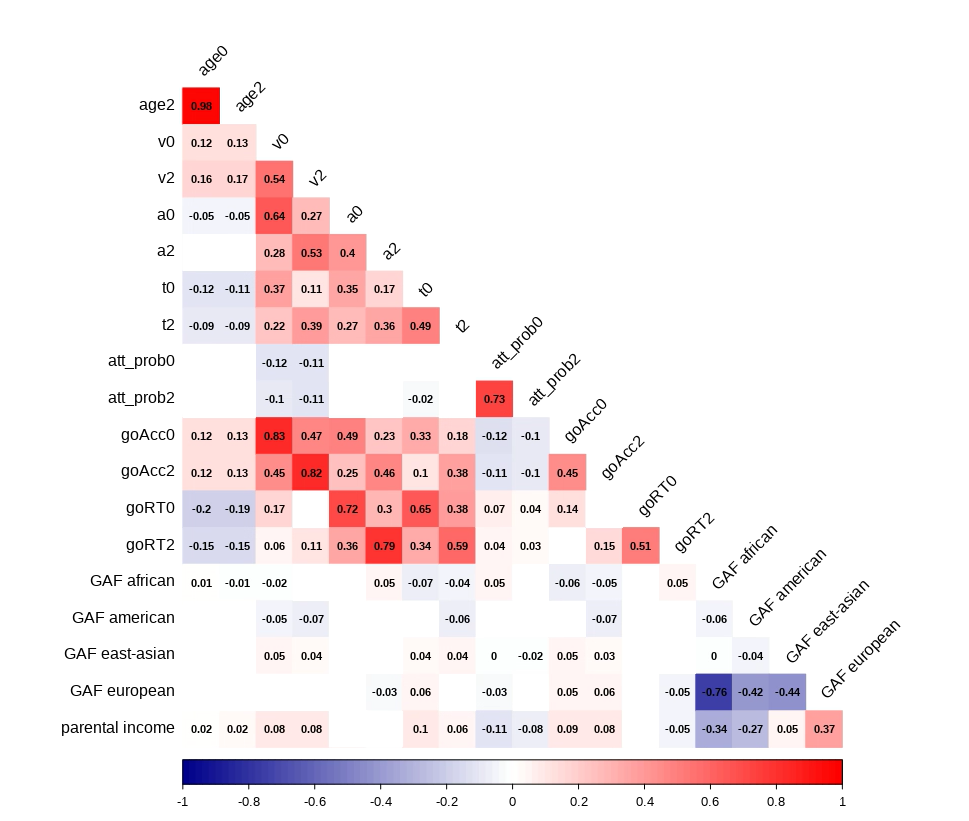
*

**Figure S12.** Correlations between behavioural variables for the males. Correlation plot showing the significant correlations between the variables included in our analyses. 0 = baseline measure, 2 = 2-year follow-up measure, r = Pearson’s r, p = p-value, v = drift rate, a = threshold, t = non-decision time, RT = reaction time, acc = accuracy, GAF = genetic ancestry factor.

**ULCS**

**
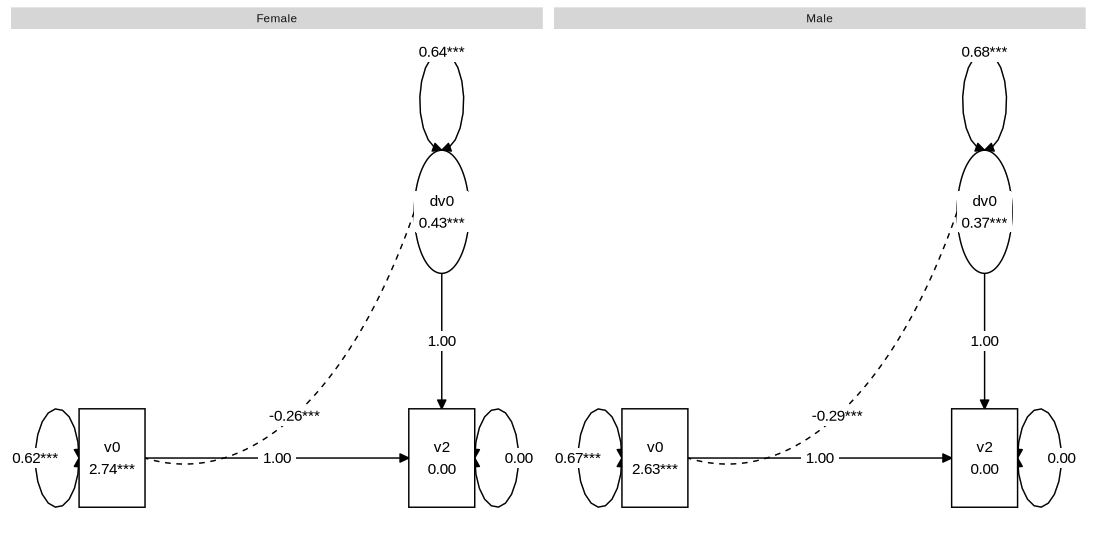
**

**Figure S13.** Graph showing results from the univariate latent change score model on drift rate (v).

**
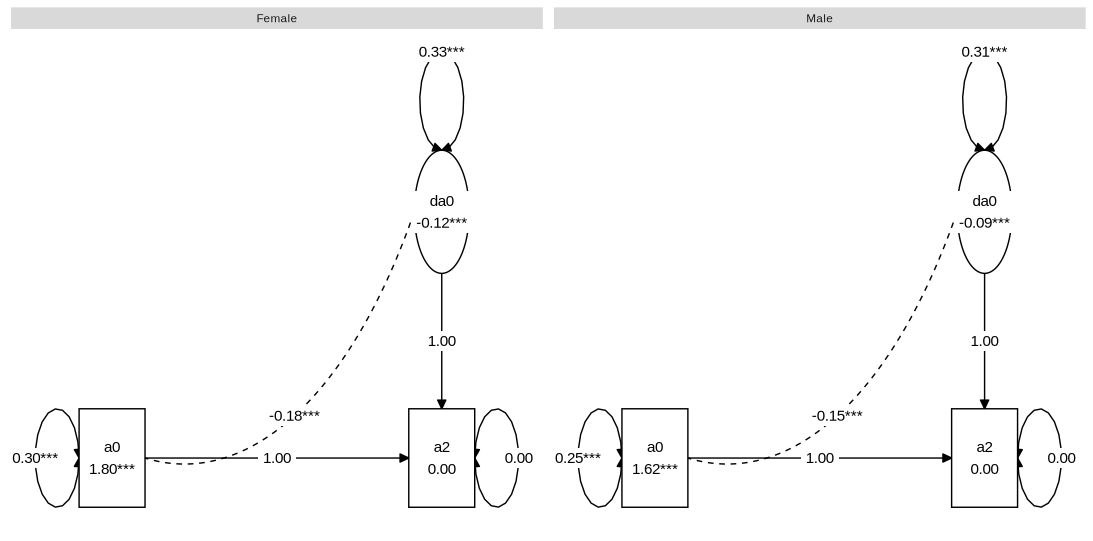
**

**Figure S14.** Graph showing results from the univariate latent change score model on decision threshold (a).

**
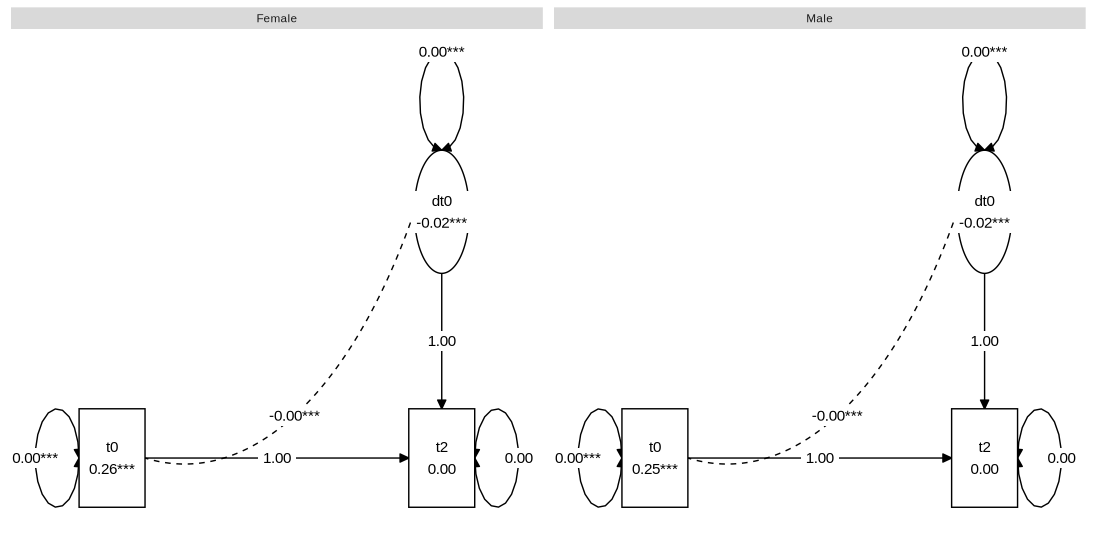
**

**Figure S15.** Graph showing results from the univariate latent change score model on non-decision time (t).

**
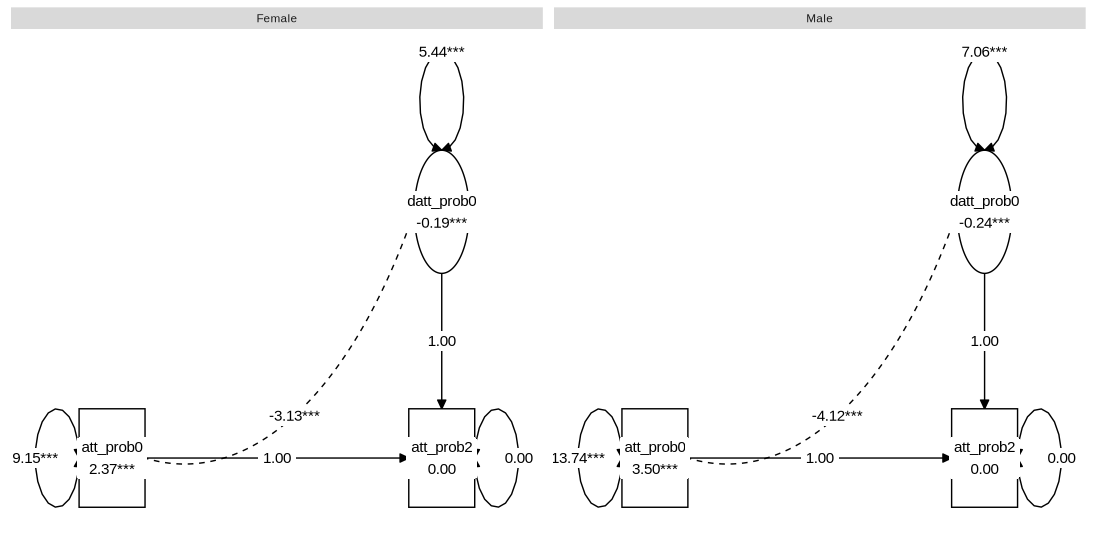
**

**Figure S16.** Graph showing results from the univariate latent change score model on attention problems (att_prob).

**Results from latent change score models with parental education as additional covariate**

As parental education is often found to correlate with child cognition, it is interesting to ask whether the current results are driven by this. We therefore performed sensitivity analyses where we added highest level of parental education as a covariate to our analyses.

**ULCS**

Model fits from the four univariate latent change score (ULCS) models are presented in table S4.

**Table S4.**Fit statistics for the multigroup ULCS models

|  | Chi square (df) | CFI | RMSEA | SRMR |
| --- | --- | --- | --- | --- |
| Drift rate | (14) 15.47 | .999 | .005 | .010 |
| Decision threshold | (14) 4.49 | 1.000 | .000 | .005 |
| Non-decision time | (14) 21.07 | .995 | .012 | .008 |
| Attention problems | (14) 18.40 | .999 | .009 | .006 |

***Drift rate (v)***

The model results are presented in Table S5**.** The model showed significant positive mean change in drift rate for both females and males, indicating that improvement in evidence accumulation rate occurs from about age 10 to age 12. There were also significant variances in baseline scores and change for both sexes. The significant negative correlation between baseline and change indicated that individuals with high initial levels of drift rate improved less.

When testing for sex differences by means of parameter constraints, the initial level of drift rate (Δχ2 (1) = 34.77, p < .001) and the latent change score (Δχ2 (1) = 7.25, p = .007) differed significantly across sexes, with higher average drift rates and a greater change score for females (estimated mean = 2.74, estimated mean change = .434) than males (estimated mean = 2.63, estimated mean change = .372). Sex differences were also found for variances of baseline scores (Δχ2 (1) = 5.15, p = .023) with higher variations across participants for males (estimate variance in baseline = .669) than females (estimated variance in baseline = .621). However, no sex differences were found in the variance of the latent change score (Δχ2 (1) = 1.77, p = .183).

**Table S5.**Results from the multigroup ULCS model on drift rate.

| Parameter | Females | | | | Males | | | |
| --- | --- | --- | --- | --- | --- | --- | --- | --- |
|  | Est. | SE | z | p | Est. | SE | z | p |
| Baseline mean | 2.739 | .013 | 203.131 | **<.001** | 2.627 | .013 | 197.229 | **<.001** |
| Mean change | .434 | .017 | 25.758 | **<.001** | .372 | .016 | 22.923 | **<.001** |
| Variance of baseline score | .621 | .015 | 41.193 | **<.001** | .669 | .015 | 43.187 | **<.001** |
| Variance of change score | .639 | .023 | 27.351 | **<.001** | .683 | .023 | 29.410 | **<.001** |
| Correlation baseline-change | -.417 | .014 | -18.221 | **<.001** | -.420 | .015 | -19.065 | **<.001** |

*Note.* Est. = unstandardised estimates except for the covariance where standardised estimates are provided, SE = standard error, z = z-score, p = p-value. Significant results are highlighted in bold.

***Decision threshold (a)***

The model results are presented in Table S6**.** The model demonstrated significant negative mean change in decision threshold for both sexes indicating a decrease in decision threshold from about age 10 to age 12. There were also significant variances in baseline scores and change for both sexes. The significant negative correlations between baseline and change indicate that those with initially high decision threshold decreased more.

When testing for sex differences by means of parameter constraints, the initial level of decision threshold differed significantly across sexes (Δχ2 (1) = 195.37, p < .001), with higher average decision threshold for females (estimated mean = 1.80) than males (estimated mean = 1.62). Sex differences were also found for the variance of the baseline scores (Δχ2 (1) = 8.45, p = .004) with higher variation across female (estimated variance = .303) than male participants (estimated variance = .252). We further found significant sex differences in mean change (Δχ2 (1) = 3.90, p = .048) with a more negative change for females (estimated change = -.12) than males (estimated change = -.09). However, no sex differences were found when constraining the variance of the latent change factor (Δχ2 (1) = .71, p = .399).

**Table S6.**Results from the multigroup ULCS model on decision threshold.

| Parameter | Females | | | | Males | | | |
| --- | --- | --- | --- | --- | --- | --- | --- | --- |
|  | Est. | SE | z | p | Est. | SE | z | p |
| Baseline mean | 1.799 | .010 | 189.166 | **<.001** | 1.619 | .008 | 195.728 | **<.001** |
| Mean change | -.117 | .012 | -9.930 | **<.001** | -.086 | .011 | -7.989 | **<.001** |
| Variance of baseline score | .303 | .014 | 22.243 | **<.001** | .252 | .011 | 22.411 | **<.001** |
| Variance of change score | .332 | .017 | 19.625 | **<.001** | .312 | .016 | 18.958 | **<.001** |
| Correlation baseline-change | -.576 | .013 | -14.384 | **<.001** | -.530 | .010 | -14.381 | **<.001** |

*Note.* Est. = unstandardised estimates except for covariance where standardised estimates are provided

***Non-decision time (t)***

The model results are presented in Table S7**.** The model demonstrated significant negative mean change in non-decision time for both sexes, indicating a reduction in time spent on non-decision processes from about age 10 to age 12. There were also significant variances in baseline scores and change for both sexes. The negative correlation between baseline and change shows that those with initial high scores decreased more.

When testing for sex differences by means of parameter constraints, the baseline non-decision time differed significantly across sexes (Δχ2 (1) = 84.17, p < .001), with longer non-decision time for females (estimated mean = .264) than males (estimated mean = .255). Sex differences were also found when constraining the variance of the baseline scores (Δχ2 (1) = 5.51, p = .019) and the variance of the latent change factor (Δχ2 (1) = 6.45, p = .011) with higher variation across participants for males (estimated baseline variance = .002 (z-score = 40.89), estimated variance in change = .002 (z-score = 29.08)) than females (estimated baseline variance = .002 (z-score = 37.56), estimated variance in change = .002 (z-score = 27.20). Lastly, constraining the mean level of latent change did not suggest sex differences (Δχ2 (1) = 1.28, p = .258).

**Table S7.**Results from the multigroup ULCS model on non-decision time.

| Parameter | Females | | | | Males | | | |
| --- | --- | --- | --- | --- | --- | --- | --- | --- |
|  | Est. | SE | z | p | Est. | SE | z | p |
| Baseline mean | .264 | .001 | 349.918 | **<.001** | .255 | .001 | 341.669 | **<.001** |
| Mean change | .-.020 | .001 | -23.581 | **<.001** | -.021 | .001 | -25.926 | **<.001** |
| Variance of baseline score | .002 | .000 | 37.564 | **<.001** | .002 | .000 | 40.888 | **<.001** |
| Variance of change score | .002 | .000 | 27.196 | **<.001** | .002 | .000 | 29.081 | **<.001** |
| Correlation baseline-change | -.592 | .000 | -22.763 | **<.001** | -.645 | .000 | -26.653 | **<.001** |

*Note.* Est. = unstandardised estimates except for covariance where standardised estimates are provided, SE = standard error, z = z-score, p = p-value. Significant results are highlighted in bold.

***Attention problems***

The model results are presented in Table S8**.** The model demonstrated significant negative mean change in attention problems for both males and females, indicating a reduction in attention problems from about age 10 to age 12. There were also significant variances in baseline scores and change scores for both sexes. The significant negative correlation between baseline and change indicates that individuals with high initial levels of attention problems tended to decrease more.

When testing for sex differences by means of parameter constraints, the baseline level of attention problems differed significant across sexes (Δχ2 (1) = 211.62, p < .001), with higher average attention problems for males (estimated mean = 3.51) than females (estimated mean = 2.38). Sex differences were also found for variances of baseline scores (Δχ2 (1) = 77.69, p < .001) and the variance of the latent change factor (Δχ2 (1) = 21.51, p < .001), with higher variations across participants for males (estimated variance in baseline scores = 13.74, estimated variance in change score = 7.06) than females (estimated variance in baseline score = 9.13, estimated variance in change score = 5.44). However, no sex differences in the mean level of latent change were found: Δχ2 (1) = .07, p = .416.

**Table S8.**Results from multigroup ULCS model on attention problems

| Parameter | Females | | | | Males | | | |  |
| --- | --- | --- | --- | --- | --- | --- | --- | --- | --- |
|  | Est. | SE | z | p | Est. | SE | z | p | |
| Baseline mean | 2.377 | .050 | 47.772 | **<.001** | 3.507 | .057 | 60.997 | **<.001** | |
| Mean change | -.193 | .045 | -4.336 | **<.001** | -.246 | .048 | -5.155 | **<.001** | |
| Variance of baseline score | 9.131 | .330 | 27.699 | **<.001** | 13.739 | .379 | 36.233 | **<.001** | |
| Variance of change score | 5.440 | .234 | 23.264 | **<.001** | 7.062 | .257 | 27.439 | **<.001** | |
| Correlation baseline-change | -.444 | .214 | -14.602 | **<.001** | -.418 | .245 | -16.825 | **<.001** | |

*Note.* Est. = unstandardised estimates except for covariance where standardised estimates are provided, SE = standard error, z = z-score, p = p-value. Significant results are highlighted in bold.

**BLCS**

Model fit statistics for the three BLCS models are presented in table S9 while the results are presented in table S10.

**Table S9.**Fit statistics for the multigroup BLCS models.

|  | Chi square (df) | CFI | RMSEA | SRMR |
| --- | --- | --- | --- | --- |
| Drift rate | (28) 33.56 | .999 | .007 | .009 |
| Decision threshold | (28) 26.822 | 1.000 | .000 | .007 |
| Non-decision time | (28) 41.56 | .997 | .011 | .009 |

**Table S10.**Results from BLCS models for each DDM parameter where numbers 1-4 represent the effects of interest: (1) correlation at baseline, (2) regression (attention problems at baseline related to change in DDM parameter), (3) regression (DDM parameter at baseline related to change in attention problems), (4) correlation of change score.

|  | Parameter | Females | | | | Males | | | |
| --- | --- | --- | --- | --- | --- | --- | --- | --- | --- |
|  |  | Est. | SE | z | p | Est. | SE | z | p |
| Drift rate (v) |  |  |  |  |  |  |  |  |  |
| 1 | att0↔v0 (ϕ) | -.099 | .042 | -5.598 | **<.001** | -.113 | .052 | -6.599 | **<.001** |
| 2 | att0→Δv (γ_1_) | -.061 | .005 | -2.954 | **.003** | -.051 | .004 | -2.726 | **.006** |
| 3 | v0→Δatt (γ_2_) | -.084 | .055 | -4.416 | **<.001** | -.033 | .058 | -1.841 | .066 |
| 4 | Δatt↔Δv (ρ) | -.085 | .035 | -3.705 | **<.001** | -.061 | .038 | -2.866 | **.004** |
| Threshold (a) |  |  |  |  |  |  |  |  |  |
| 1 | att0↔a0 (ϕ) | -.016 | .029 | -.897 | .370 | -.002 | .032 | -.145 | .885 |
| 2 | att0→Δa (γ_1_) | .003 | .003 | .162 | .871 | -.012 | .003 | -.672 | .502 |
| 3 | a0→Δatt (γ_2_) | -.019 | .079 | -1.042 | .298 | -.010 | .100 | -.525 | .599 |
| 4 | Δatt↔Δa (ρ) | -.038 | .020 | -1.916 | .055 | -.005 | .027 | -.210 | .834 |
| Non-decision time (t) |  |  |  |  |  |  |  |  |  |
| 1 | att0↔t0 (ϕ) | -.016 | .002 | -.892 | .372 | -.013 | .003 | -.772 | .440 |
| 2 | att0→Δt (γ_1_) | .016 | .000 | .838 | .402 | -.008 | .000 | -.509 | .611 |
| 3 | t0→Δatt (γ_2_) | -.044 | 1.007 | -2.311 | **.021** | -.021 | 1.064 | -1.155 | .248 |
| 4 | Δatt↔Δt (ρ) | -.033 | .002 | -1.430 | .153 | -.027 | .002 | -1.309 | .191 |

**Note.** 0 = baseline measure, est. = estimates, SE = standard error, z = z-score, p = p-value, Δ = change. All reported effects are standardised. Statistically significant findings are highlighted in bold.

***Drift rate (v)***

First, a multigroup BLCS model for attention problems and drift rate was modelled. Inspection of the four parameters of interest, reflecting the four possible attention problems-drift rate relationships (see Table S10), showed a statistically significant negative correlation between attention problems and drift rate at baseline for both males (r = -.11, p < .001) and females (r = -.10, p < .001). The results also showed statistically significant associations of correlated change for both males (r = -.06, p = .004) and females (r = -.09, p < .001). The negative correlations indicate that those with greater increase in drift rate over time showed a greater decrease in attention problems. There were additionally coupling effects in which attention problems at baseline predicted change in drift rate for males (β=-.05, p=.006) and females (β = -.06, p=.003), and drift rate at baseline predicted change in attention problems for females (β=-.08, p<.001) but not males (β=-.03, p=.066). Next, χ^2^-difference testing for sex differences in the coefficients were carried out. Here, no significant difference between males and females were found for any of the four attention problems-drift rate relationships.

***Decision threshold (a)***

Second, a BLCS model for attention problems and decision threshold was modelled. Inspection of the four parameters of interest, reflecting the four possible attention problems-decision threshold relationships (see Table S10), showed no statistically significant correlation between attention problems and decision threshold at baseline for males or females. The results also showed no statistically significant associations of correlated change. Lastly, there were no significant coupling effects. Moreover, χ^2^-difference tests indicated no significant difference between males and females were found for any of the four attention problems-decision threshold relationships.

***Non-decision time (t)***

Third, a BLCS model for attention problems and non-decision time was. Inspection of the four parameters of interest, reflecting the four possible attention problems-non-decision time relationships (see Table S10), showed no statistically significant correlation between attention problems and non-decision time at baseline for males or females. The results further showed no statistically significant associations of correlated change. Lastly, there were no significant coupling effects except for non-decision time at baseline predicting change in attention problems for females (β=-.04, p=.021). Next, χ^2^-difference testing for sex differences were carried out. Here, no significant difference between males and females were found for any of the four attention problems-non-decision time relationships.

**Results from latent change score models with EZ-diffusion model parameters**

In the main analysis the latent change score models were performed with drift-diffusion parameters derived from HDDM. However, there are concerns around entering individual-level parameter estimates from hierarchical Bayesian model into subsequent null-hypothesis testing analyses as this can potentially bias the test statistics (Boehm et al., 2018; Evans & Wagenmakers, 2019). While separating the sample into 80 groups for the HDDM parameter estimation and the large sample size may have ameliorated the concern, it is difficult to say for sure. We therefore performed sensitivity analyses using EZ-diffusion model parameters instead of HDDM parameters.

Note that as attention problems scale was same as main analyses, a new ULCS for attention problems was not performed. See main text for results from this analysis.

**ULCS**

Model fits from the four univariate latent change score (ULCS) models are presented in table S11.

**Table S11.**Fit statistics for the multigroup ULCS models.

|  | Chi square (df) | CFI | RMSEA | SRMR |
| --- | --- | --- | --- | --- |
| Drift rate | (12) 18.01 | .997 | .011 | .011 |
| Decision threshold | (12) 10.90 | 1.000 | .000 | .008 |
| Non-decision time | (12) 24.19 | .992 | .017 | .012 |

***Drift rate (v)***

The model results are presented in Table S12**.** The model showed significant positive mean change in drift rate for both females and males, indicating that improvement in evidence accumulation rate occurs from about age 10 to age 12. There were also significant variances in baseline scores and change for both sexes. The significant negative correlation between baseline and change indicated that individuals with high initial levels of drift rate improved less.

When testing for sex differences by means of parameter constraints, the initial level of drift rate (Δχ2 (1) = 25.52, p < .001) and the latent change score (Δχ2 (1) = 14.93, p < .001) differed significantly across sexes, with higher average drift rates and a greater change score for females (estimated mean = 2.18, estimated mean change = .337) than males (estimated mean = 2.12, estimated mean change = .274). Sex differences were also found for variances of baseline scores (Δχ2 (1) = 32.39, p < .001) and variances of the latent change score (Δχ2 (1) = 12.92, p < .001) with higher variations across participants for males (estimate variance in baseline = .315, estimated variance in latent change score = .343) than females (estimated variance in baseline = .261, estimated variance in latent change score = .287).

These analyses yielded comparable results to those with HDDM parameters (see main text). However, the current model with EZ-parameters identified significant sex differences in the variance of the latent change score whereas the model with HDDM-parameters did not.

**Table S12.**Results from the multigroup ULCS model on drift rate.

| Parameter | Females | | | | Males | | | |
| --- | --- | --- | --- | --- | --- | --- | --- | --- |
|  | Est. | SE | z | p | Est. | SE | z | p |
| Baseline mean | 2.182 | .009 | 248.845 | **<.001** | 2.117 | .009 | 231.860 | **<.001** |
| Mean change | .337 | .011 | 20.420 | **<.001** | .274 | .012 | 23.766 | **<.001** |
| Variance of baseline score | .261 | .006 | 41.015 | **<.001** | .315 | .007 | 45.443 | **<.001** |
| Variance of change score | .287 | .010 | 27.484 | **<.001** | .343 | .012 | 23.766 | **<.001** |
| Correlation baseline-change | -.348 | .006 | -15.296 | **<.001** | -.387 | .007 | -17.954 | **<.001** |

*Note.* Est. = unstandardised estimates except for the covariance where standardised estimates are provided, SE = standard error, z = z-score, p = p-value. Significant results are highlighted in bold.

***Decision threshold (a)***

The model results are presented in Table S13**.** The model demonstrated significant negative mean change in decision threshold for both sexes indicating a decrease in decision threshold from about age 10 to age 12. There were also significant variances in baseline scores and change for both sexes. The significant negative correlations between baseline and change indicate that those with initially high decision threshold decreased more.

When testing for sex differences by means of parameter constraints, the initial level of decision threshold differed significantly across sexes (Δχ2 (1) = 382.22, p < .001), with higher average decision threshold for females (estimated mean = 1.30) than males (estimated mean = 1.20). Sex differences were also found for the variance of the baseline scores (Δχ2 (1) = 13.92, p < .001) with higher variation across female (estimated variance = .044) than male participants (estimated variance = .037). However, no sex differences were found I mean change (Δχ2 (1) = 3.54, p = .060) or when constraining the variance of the latent change factor (Δχ2 (1) = 2.21, p = .137).

These sensitivity analyses with EZ-diffusion parameters yielded comparable results to those with HDDM parameters (see main text). However, the current model with EZ-parameters did not identify significant sex differences in the latent change score or for the variance of the latent change score while the model with HDDM parameters did.

**Table S13.**Results from the multigroup ULCS model on decision threshold.

| Parameter | Females | | | | Males | | | |
| --- | --- | --- | --- | --- | --- | --- | --- | --- |
|  | Est. | SE | z | p | Est. | SE | z | p |
| Baseline mean | 1.297 | .004 | 358.226 | **<.001** | 1.202 | .003 | 378.373 | **<.001** |
| Mean change | -.055 | .005 | -10.615 | **<.001** | -.042 | .005 | -9.168 | **<.001** |
| Variance of baseline score | .044 | .001 | 29.450 | **<.001** | .037 | .001 | 34.495 | **<.001** |
| Variance of change score | .064 | .003 | 22.815 | **<.001** | .058 | .002 | 24.583 | **<.001** |
| Correlation baseline-change | -.474 | .013 | -14.405 | **<.001** | -.472 | .001 | -18.330 | **<.001** |

*Note.* Est. = unstandardised estimates except for covariance where standardised estimates are provided

***Non-decision time (t)***

The model results are presented in Table S14**.** The model demonstrated significant negative mean change in non-decision time for both sexes, indicating a reduction in time spent on non-decision processes from about age 10 to age 12. There were also significant variances in baseline scores and change for both sexes. The negative correlation between baseline and change shows that those with initial high scores decreased more.

When testing for sex differences by means of parameter constraints, the baseline non-decision time differed significantly across sexes (Δχ2 (1) = 123.47, p < .001), with longer non-decision time for females (estimated mean = .305) than males (estimated mean = .289). No sex differences were found for the other parameters (mean level of latent change: Δχ2 (1) = .09, p = .758; variance of the baseline scores: Δχ2 (1) = 1.96, p = .161; variance of the latent change factor Δχ2 (1) = 2.33, p = .127).

These sensitivity analyses with EZ-diffusion parameters yielded comparable results to those with HDDM parameters (see main text), except that the current model with EZ-parameters identified significant sex differences only in the baseline scores whereas the model with HDDM parameters also identified sex differences in the variance of the baseline scores and the variance of the latent change scores.

**Table S14.**Results from the multigroup ULCS model on non-decision time.

| Parameter | Females | | | | Males | | | |
| --- | --- | --- | --- | --- | --- | --- | --- | --- |
|  | Est. | SE | z | p | Est. | SE | z | p |
| Baseline mean | .305 | .001 | 281.464 | **<.001** | .289 | .001 | 274.484 | **<.001** |
| Mean change | -.035 | .001 | -29.876 | **<.001** | -.034 | .001 | -30.243 | **<.001** |
| Variance of baseline score | .004 | .000 | 37.110 | **<.001** | .004 | .000 | 42.280 | **<.001** |
| Variance of change score | .003 | .000 | 25.395 | **<.001** | .003 | .000 | 27.132 | **<.001** |
| Correlation baseline-change | -.602 | .000 | -23.757 | **<.001** | -.598 | .000 | -25.265 | **<.001** |

*Note.* Est. = unstandardised estimates except for covariance where standardised estimates are provided, SE = standard error, z = z-score, p = p-value. Significant results are highlighted in bold.

***BLCS***

Model fit statistics for the three BLCS models are presented in table S15 while the results are presented in table S16.

**Table S15.**Fit statistics for the multigroup BLCS models.

|  | Chi square (df) | CFI | RMSEA | SRMR |
| --- | --- | --- | --- | --- |
| Drift rate | (24) 35.08 | .998 | .011 | .010 |
| Decision threshold | (23) 27.61 | .999 | .007 | .007 |
| Non-decision time | (24) 44.29 | .996 | .015 | .011 |

**Table S16.**Results from BLCS models for each DDM parameter where numbers 1-4 represent the effects of interest: (1) correlation at baseline, (2) regression (attention problems at baseline related to change in DDM parameter), (3) regression (DDM parameter at baseline related to change in attention problems), (4) correlation of change score.

|  | Parameter | Females | | | | Males | | | |
| --- | --- | --- | --- | --- | --- | --- | --- | --- | --- |
|  |  | Est. | SE | z | p | Est. | SE | z | p |
| Drift rate (v) |  |  |  |  |  |  |  |  |  |
| 1 | att0↔v0 (ϕ) | -.116 | .028 | -6.508 | **<.001** | -.127 | .036 | -7.297 | **<.001** |
| 2 | att0→Δv (γ_1_) | -.064 | .004 | -2.998 | **.003** | -.057 | .003 | -2.892 | **.004** |
| 3 | v0→Δatt (γ_2_) | -.095 | .087 | -4.906 | **<.001** | -.031 | .087 | -1.668 | .095 |
| 4 | Δatt↔Δv (ρ) | -.083 | .025 | -3.504 | **<.001** | -.068 | .028 | -3.200 | **.001** |
| Threshold (a) |  |  |  |  |  |  |  |  |  |
| 1 | att0↔a0 (ϕ) | -.007 | .011 | -.370 | .712 | .025 | .012 | 1.478 | .140 |
| 2 | att0→Δa (γ_1_) | .019 | .002 | 1.053 | .292 | -.007 | .001 | -.427 | .669 |
| 3 | a0→Δatt (γ_2_) | -.008 | .202 | -.421 | .674 | -.019 | .265 | -.974 | .330 |
| 4 | Δatt↔Δa (ρ) | -.008 | .010 | -.864 | .388 | .016 | .011 | .724 | .469 |
| Non-decision time (t) |  |  |  |  |  |  |  |  |  |
| 1 | att0↔t0 (ϕ) | .009 | .003 | -499 | .618 | .007 | .004 | .405 | .685 |
| 2 | att0→Δt (γ_1_) | .011 | .000 | .607 | .544 | -.012 | .000 | -.713 | .476 |
| 3 | t0→Δatt (γ_2_) | -.027 | .718 | -1.371 | .170 | -.003 | .757 | -.148 | .882 |
| 4 | Δatt↔Δt (ρ) | -.033 | .002 | -1.413 | .158 | -.024 | .002 | -1.103 | .270 |

**Note.** 0 = baseline measure, est. = estimates, SE = standard error, z = z-score, p = p-value, Δ = change. All reported effects are standardised. Statistically significant findings are highlighted in bold.

***Drift rate (v)***

First, a multigroup BLCS model for attention problems and drift rate was modelled. Inspection of the four parameters of interest, reflecting the four possible attention problems-drift rate relationships (see Table S16), showed a statistically significant negative correlation between attention problems and drift rate at baseline for both males (r = -.13, p < .001) and females (r = -.12, p < .001). The results also showed statistically significant associations of correlated change for both males (r = -.07, p = .004) and females (r = -.08, p < .001). The negative correlations indicate that those with greater increase in drift rate over time showed a greater decrease in attention problems. There were additionally coupling effects in which attention problems at baseline predicted change in drift rate for males (β=-.06, p=.006) and females (β = -.06, p=.003), and drift rate at baseline predicted change in attention problems for females (β=-.10, p<.001) but not males (β=-.03, p=.095). Next, χ^2^-difference testing for sex differences in the coefficients were carried out and results showed significant sex differences in the coupling between baseline drift rate and change in attention problems (χ^2^ (1) = 5.41, p = .020) with females showing a much stronger relationship than males. No other significant differences were found between males and females.

As with the ULCS models, these sensitivity analyses with EZ-diffusion parameters yielded comparable results to the main analyses with HDDM parameters (see main text). Note however, that in the EZ analyses there was significant sex differences in the coupling effect of baseline drift rate predicting change in attention problems whereas this sex difference was non-significant in the main analyses.

***Decision threshold (a)***

Second, a BLCS model for attention problems and decision threshold was modelled. Inspection of the four parameters of interest, reflecting the four possible attention problems-decision threshold relationships (see Table S16), showed no statistically significant correlation between attention problems and decision threshold at baseline for males or females. The results also showed no statistically significant associations of correlated change. Lastly, there were no significant coupling effects. Moreover, χ^2^-difference tests indicated no significant difference between males and females were found for any of the four attention problems-decision threshold relationships. These sensitivity analyses with EZ-diffusion parameters gave similar results to the main analyses with HDDM parameters.

***Non-decision time (t)***

Third, a BLCS model for attention problems and non-decision time was. Inspection of the four parameters of interest, reflecting the four possible attention problems-non-decision time relationships (see Table S16), showed no statistically significant correlation between attention problems and non-decision time at baseline for males or females. The results further showed no statistically significant associations of correlated change. Lastly, there were no significant coupling effects. Next, χ^2^-difference testing for sex differences were carried out. Here, no significant difference between males and females were found for any of the four attention problems-non-decision time relationships.

In these sensitivity analyses with EZ-diffusion parameters, the results were mainly identical, however the significant coupling effect for females with baseline non-decision time predicting change in attention problems in the main analyses was not significant in the sensitivity analyses.

**Results from latent change score models with ADHD subscale**

In the main analysis the latent change score models were performed with the attention problems subscale from the Child Behaviour Checklist. However, as some items fit less well with ADHD symptoms a sensitivity analysis with the ADHD subscale was performed.

Note that as the DDM parameters were the same as in the main analyses, new ULCS models for these parameters were not performed. See main text for results on ULCS on HDDM parameters.

**ULCS: attention problems measured by ADHD subscale**

The multigroup ULCS model for attention problems fit the data well: χ^2^ (12) = 16.12, CFI = .999, RMSEA = .009, and SRMR = .007. The model results are presented in Table S17. The model demonstrated significant negative mean change in attention problems for both males and females, indicating a reduction in attention problems from about age 10 to age 12. There were also significant variances in baseline scores and change scores for both sexes. The significant negative correlation between baseline and change indicates that individuals with high initial levels of attention problems tended to decrease more.

When testing for sex differences by means of parameter constraints, the baseline ADHD score differed significantly across sexes (Δχ2 (1) = 200.47, p < .001), with ADHD scores for males (estimated mean = 3.062) than females (estimated mean = 2.123). Sex differences were also found for variances of baseline scores (Δχ2 (1) = 71.35, p < .001) and the variance of the latent change factor (Δχ2 (1) = 15.53, p < .001), with higher variations across participants for males (estimated variance in baseline scores = 9.82, estimated variance in change score = 5.11) than females (estimated variance in baseline score = 6.83, estimated variance in change score = 4.08). However, no sex differences in the mean level of latent change were found: Δχ2 (1) = .94, p = .333.

**Table S17.**Results from the multigroup ULCS model on ADHD.

| Parameter | Females | | | | Males | | | |
| --- | --- | --- | --- | --- | --- | --- | --- | --- |
|  | Est. | SE | z | p | Est. | SE | z | p |
| Baseline mean | 2.123 | .043 | 49.497 | **<.001** | 3.062 | .048 | 63.148 | **<.001** |
| Mean change | -.273 | .038 | -7.141 | **<.001** | -.326 | .040 | -8.101 | **<.001** |
| Variance of baseline score | 6.834 | .228 | 29.929 | **<.001** | 9.818 | .245 | 40.101 | **<.001** |
| Variance of change score | 4.078 | .177 | 23.005 | **<.001** | 5.106 | .186 | 27.430 | **<.001** |
| Correlation baseline-change | -.472 | .159 | -15.666 | **<.001** | -.434 | .165 | -18.657 | **<.001** |

*Note.* Est. = unstandardised estimates except for covariance where standardised estimates are provided, SE = standard error, z = z-score, p = p-value. Significant results are highlighted in bold.

**BLCS**

Model fit statistics for the three BLCS models are presented in table S18 while the results are presented in table S19.

**Table S18.**
Fit statistics for the multigroup BLCS models.

|  | Chi square (df) | CFI | RMSEA | SRMR |
| --- | --- | --- | --- | --- |
| Drift rate | (24) 31.05 | .999 | .09 | .010 |
| Decision threshold | (23) 23.93 | 1.000 | .003 | .008 |
| Non-decision time | (24) 36.68 | .998 | .012 | .010 |

**Table S19.**Results from BLCS models for each DDM parameter where numbers 1-4 represent the effects of interest: (1) correlation at baseline, (2) regression (attention problems at baseline related to change in DDM parameter), (3) regression (DDM parameter at baseline related to change in attention problems), (4) correlation of change score.

|  | Parameter | Females | | | | Males | | | |
| --- | --- | --- | --- | --- | --- | --- | --- | --- | --- |
|  |  | Est. | SE | z | p | Est. | SE | z | p |
| Drift rate (v) |  |  |  |  |  |  |  |  |  |
| 1 | att0↔v0 (ϕ) | -.108 | .037 | -6.097 | **<.001** | -.106 | .044 | -6.136 | **<.001** |
| 2 | att0→Δv (γ_1_) | -.067 | .006 | -3.307 | **.001** | -.045 | .005 | -2.405 | **.016** |
| 3 | v0→Δatt (γ_2_) | -.070 | .046 | -3.810 | **<.001** | -.035 | .050 | -1.921 | .055 |
| 4 | Δatt↔Δv (ρ) | -.083 | .031 | -3.469 | **.001** | -.057 | .032 | -2.745 | **.006** |
| Threshold (a) |  |  |  |  |  |  |  |  |  |
| 1 | att0↔a0 (ϕ) | -.029 | .024 | -1.703 | .089 | -.009 | .027 | -.544 | .587 |
| 2 | att0→Δa (γ_1_) | -.006 | .004 | -.345 | .730 | -.009 | .003 | -.551 | .582 |
| 3 | a0→Δatt (γ_2_) | -.016 | .064 | -.911 | .362 | -.013 | .084 | -.712 | .476 |
| 4 | Δatt↔Δa (ρ) | -.031 | .018 | -1.475 | .140 | .009 | .024 | .374 | .708 |
| Non-decision time (t) |  |  |  |  |  |  |  |  |  |
| 1 | att0↔t0 (ϕ) | .009 | .003 | -499 | .618 | -.020 | .002 | -1.176 | .240 |
| 2 | att0→Δt (γ_1_) | .011 | .000 | .607 | .544 | -.021 | .000 | -1.444 | .149 |
| 3 | t0→Δatt (γ_2_) | -.027 | .718 | -1.371 | .170 | -.022 | .900 | -1.221 | .222 |
| 4 | Δatt↔Δt (ρ) | -.033 | .002 | -1.413 | .158 | -.033 | .001 | -1.592 | .111 |

**Note.** 0 = baseline measure, est. = estimates, SE = standard error, z = z-score, p = p-value, Δ = change. All reported effects are standardised. Statistically significant findings are highlighted in bold.

***Drift rate (v)***

First, a multigroup BLCS model for attention problems and drift rate was modelled. Inspection of the four parameters of interest, reflecting the four possible attention problems-drift rate relationships (see Table SX), showed a statistically significant negative correlation between attention problems and drift rate at baseline for both males (r = -.11, p < .001) and females (r = -.11, p < .001). The results also showed statistically significant associations of correlated change for both males (r = -.06, p = .004) and females (r = -.08, p < .001). The negative correlations indicate that those with greater increase in drift rate over time showed a greater decrease in attention problems. There were additionally coupling effects in which attention problems at baseline predicted change in drift rate for males (β=-.05, p=.016) and females (β = -.07, p=.001), and drift rate at baseline predicted change in attention problems for females (β=-.07, p<.001) but not males (β=-.04, p=.055). Next, χ^2^-difference testing for sex differences in the coefficients were carried out. Here, no significant difference between males and females were found for any of the four attention problems-drift rate relationships.

***Decision threshold (a)***

Second, a BLCS model for attention problems and decision threshold was modelled. Inspection of the four parameters of interest, reflecting the four possible attention problems-decision threshold relationships (see Table SX), showed no statistically significant correlation between attention problems and decision threshold at baseline for males or females. The results also showed no statistically significant associations of correlated change. Lastly, there were no significant coupling effects. Moreover, χ^2^-difference tests indicated no significant difference between males and females were found for any of the four attention problems-decision threshold relationships.

***Non-decision time (t)***

Third, a BLCS model for attention problems and non-decision time was. Inspection of the four parameters of interest, reflecting the four possible attention problems-non-decision time relationships (see Table SX), showed no statistically significant correlation between attention problems and non-decision time at baseline for males or females. The results further showed no statistically significant associations of correlated change. Lastly, there were no significant coupling effects. Next, χ^2^-difference testing for sex differences were carried out. Here, no significant difference between males and females were found for any of the four attention problems-non-decision time relationships.
